# Variability of the arterial input function in small-animal dynamic PET imaging

**DOI:** 10.1186/s13550-025-01367-8

**Published:** 2026-02-11

**Authors:** Samuel Kuttner, Rodrigo Berzaghi, Laurence Convert, Otman Sarrhini, Roger Lecomte, Rune Sundset

**Affiliations:** 1 The PET Imaging Center, University Hospital of North Norway, Tromsø, Norway; 2 UiT Machine Learning Group, Department of Physics and Technology, UiT The Arctic University of Norway, Tromsø, Norway; 3 Nuclear Medicine and Radiation Biology Research Group, Department of Clinical Medicine, UiT The Arctic University of Norway, Tromsø, Norway; 4 Sherbrooke Molecular Imaging Centre of CRCHUS and Department of Medical Imaging and Radiation Sciences, Université de Sherbrooke, Sherbrooke, QC, Canada; 5 Imaging Research & Technology Inc., Sherbrooke, Canada

**Keywords:** Dynamic positron emission tomography (PET), Small-animal PET, Arterial input function, Variability, Repeatability

## Abstract

**Background:**

Dynamic positron emission tomography (PET) combined with tracer kinetic modeling enables non-invasive quantification of biochemical processes. A prerequisite is availability of the arterial input function (AIF), which, in small-animal PET imaging, involves labor-intensive terminal surgery. Deep learning based input function (DLIF) allows estimation of whole-blood tracer concentration from PET data, avoiding arterial cannulation, but require comprehensive validation. The aim of this study was to collect dynamic PET and AIF data under controlled conditions and to evaluate the variability of AIF, image-derived input function (IDIF), and kinetic modeling parameters, which is important for future DLIF model training. Dynamic PET and AIF data were collected prospectively from 112 mice in groups with varying experimental conditions, including radiotracer injection volume, injection time, withdrawal rate, mouse age, strain, radiopharmaceutical, and PET scanner. Brain, myocardium, left ventricle and liver were delineated for kinetic modeling and IDIF generation. Curve features and kinetic modeling parameters were computed, using both AIF and IDIF, and compared across groups using box plots and statistical tests. Intra-subject repeatability was evaluated in six mice using three small-volume radiotracer injections.

**Results:**

Experimental factors such as mouse strain, injection time, withdrawal rate, PET scanner and radiopharmaceutical significantly affect AIF and IDIF shapes, while injection volume and mouse age, did not introduce bias. AIF measurements within the same subject were highly repeatable.

**Conclusions:**

This study collected a comprehensive dataset of dynamic PET and AIF measurements under controlled conditions to evaluate the variability of AIF, IDIF, and kinetic modeling parameters. The findings provide valuable insights into input function variability, with potential implications for the future development of DLIF models across diverse experimental conditions.

**Supplementary Information:**

The online version contains supplementary material available at 10.1186/s13550-025-01367-8.

## Background

Positron emission tomography (PET) is a molecular imaging method that provides non-invasive absolute quantification of biochemical processes like glucose metabolism [1]. This is accomplished by dynamic PET imaging combined with tracer kinetic modeling. A prerequisite is the availability of the tracer uptake curve in both tissue and in arterial blood, the latter know as the arterial input function (AIF) [2]. In small-animal PET imaging, arterial cannulation is a labor-intensive, terminal procedure in mice, hampering routine use and preventing longitudinal studies for monitoring disease progression or therapy response [3, 4]. Alternative methods have been proposed to overcome these shortcomings, including population-based AIF [5], image-derived input function (IDIF) [6], and simultaneous estimation [7, 8], all which have limitations. Recently, we proposed deep learning based input function (DLIF) for estimation of the whole-blood tracer concentration directly from dynamic $\mathrm {[^{18}F]FDG}$ PET data [9–11]. While the initial DLIF model was developed using IDIF labels [9, 10], we have also validated its applicability on a small dataset with arterial blood sample labels [11] and more recently trained a DLIF model entirely using AIF labels [12]. Supervised deep learning models are trained using iterative update of the model parameters to minimize the error between predictions and ground truth labels [13]. For DLIF, the dynamic PET data are used as input, while the AIF curves are the labels, enabling the model to learn a mapping from PET imaging features to arterial tracer concentration. In small-animal dynamic PET imaging, it is well known that radiotracer injection rate [2], arterial blood withdrawal rate [14, 15] and catheter lengths [14] have a direct impact on the shape of the measured AIF. Also, factors such as mouse strain [16], age [17], dietary state [18, 19], body temperature [20], anesthesia [21] and radiotracer injection volume [22] significantly influence animal physiology and, consequently, also the tracer uptake. However, it is less studied how variables such as radiotracer injection volume, injection time, mouse age, strain and radiopharmaceutical, affects the shape of the input function, which is crucial for understanding the implications of data variability for DLIF model training. For instance, a large variability in the labels, or input data, between experimental condition A and B likely means that a DLIF model trained under condition A may not generalize to condition B, requiring the collection of a new dataset for the latter. In order to facilitate future validation of the DLIF method, we here collect a state-of-the-art benchmarking dataset of small-animal dynamic PET images coupled with AIF data where we systematically varied experimental conditions that could impact the input imaging data or the shape of the AIF labels. The DLIF model likely, but not necessarily, uses image regions similar to the AIF to map the input data to the predictions. Therefore, the aim of the current work was to investigate the variability of the measured AIF and an estimated IDIF. In addition, we present reference kinetic modeling parameters under the various experimental conditions.

## Methods

### Animal imaging and blood sampling

**Fig. 1:**
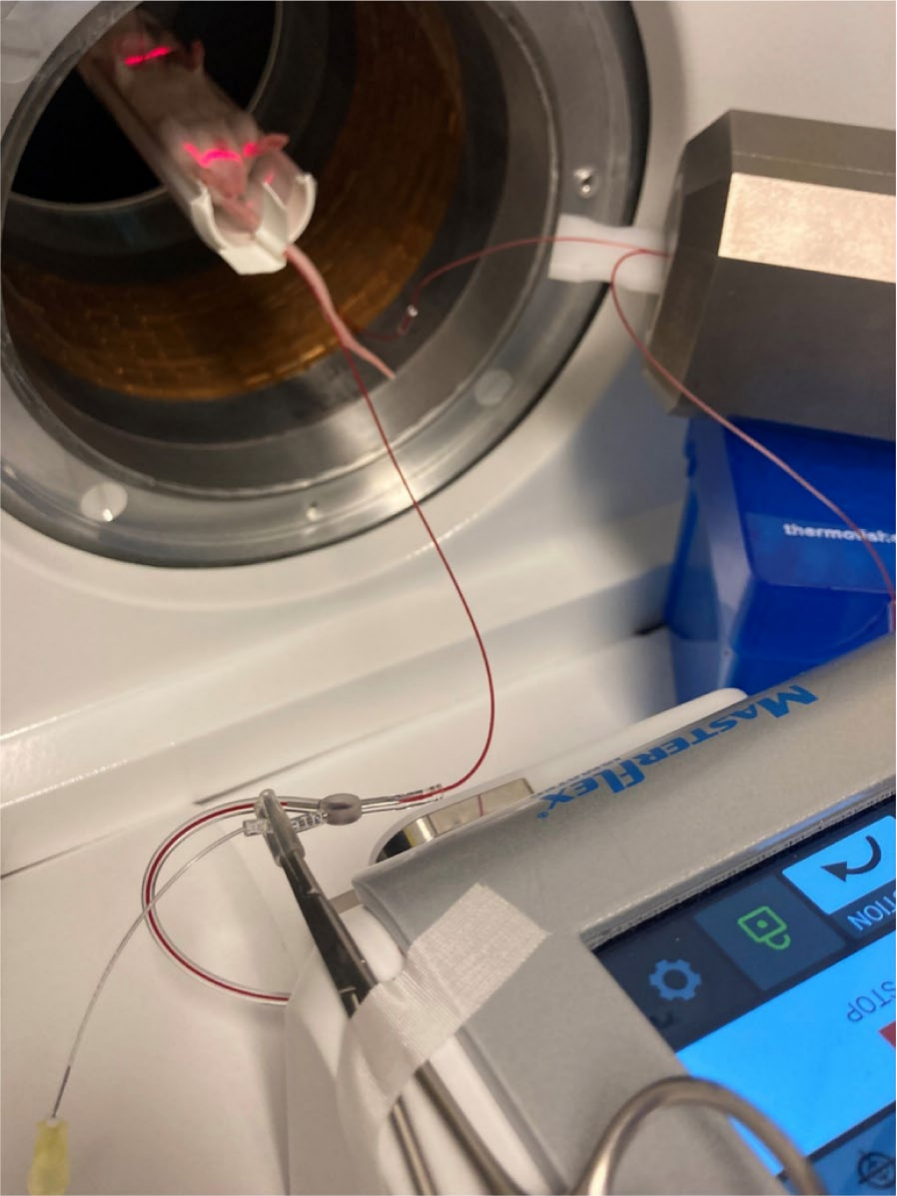
Photo of the experimental setup showing a mouse in the PET/CT scanner (upper left), the arterial line passing through the radiation detector (upper right), the peristaltic pump (lower right), and the injection catheter going into the Y-connector (lower left)

The small-animal imaging data from 112 healthy female mice were included prospectively at two different research institutions, UiT The Arctic University of Norway (UiT) and Université de Sherbrooke (UdS) (Table 1). The data were acquired during multiple PET imaging sessions between January 2023 and January 2025, with 1–3 mice scanned during each session. The full experimental details are given in Supplementary Section S1 through S6. Briefly, an arterial-venous shunt was created between the carotid artery and the tail vein of the mouse, to allow continuous arterial line measurements during the entire PET scan without excessive blood loss (Fig. 1). Three manual blood samples were collected late in the scan to allow calibration of the continuous arterial line measurements and to determine the actual arterial withdrawal rate for each mouse. To improve visualization of noisy AIF curves and account for timing delay, a parametric model was applied to the calibrated AIF data [23]. Supplementary Figure S2 illustrates a measured and calibrated AIF alongside the original and delay-corrected parametric fit. Nine mice scanned at UiT defined the reference group, consisting of 9-week-old Balb/cJRj mice injected with 15 MBq $\mathrm {[^{18}F]FDG}$ in 100 $\mathrm {\mu l}$ saline over 30 s, with continuous arterial blood withdrawal at a rate of 120 $\mathrm {\mu l/min}$ and simultaneous dynamic PET/computed tomography (CT) scanning for 45.5 minutes. Following the reference experiments, seven additional experiments were conducted, where one variable was varied at a time, to investigate its influence on the AIF, IDIF, and on tracer kinetic modeling. The following variables were considered: radiotracer injection volume, radiotracer injection time, arterial blood withdrawal rate, mouse age, mouse strain, radiopharmaceutical, and PET scanner. In addition the intra-subject repeatability of the AIF was evaluated by performing three small-volume radiotracer injections in the same mouse during the same imaging session. The number of mice included in each experiment group was between 4–9 (Table 1).

**Table 1:** Overview of variables in each experiment

Variable	Value	N	PET facility
Injection volume$\mathrm {[\mu l]}$	30 **100** 150	6 9$^{\dag }$ 6	UiT
Injection time$\mathrm {[s]}$	15 **30** 60	6 9$^{\dag }$ 6	UiT
Withdrawal rate$\mathrm {[\mu l/min]}$	30 60 **120**	6 6 9$^{\dag }$	UiT
Age$\mathrm {[weeks]}$	**9** 12 16 20 24	9$^{\dag }$ 6 6 6 6	UiT
Strain	**Balb/cJRj** Balb/cJ Balb/cAnNCrl C57BL/6JRj	9$^{\dag }$ 5 7 8	UiT UdS UiT UiT
Radiopharmaceutical	$\mathrm {[^{18}F]FDG}$ $\mathrm {[^{18}F]FDOPA}$ $\mathrm {[^{68}Ga]PSMA}$−617	9$^{\dag }$ 6 4	UiT
PET scanner	**PET/CT** PET/MR	9$^{\dag }$ 7	UiT
Triple injection	Injection volume: 30$\mathrm { \mu l}$	6	UiT
Total N		112	

Boldface indicates reference settings. In each experiment, only the indicated variable waschanged, while the rest were kept at reference settings. Note that themice in the reference group (${\dag }$) are counted only once in the Total N

### Volume of interest delineation

**Fig. 2:**
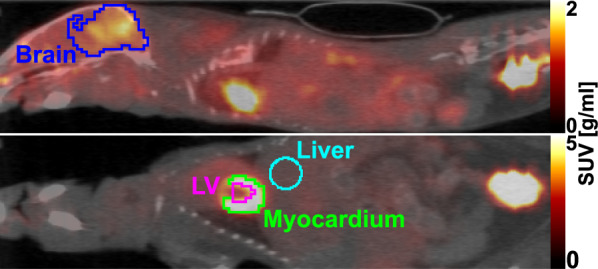
An example of the volumes of interest in one representative $\mathrm {[^{18}F]FDG}$ PET/CT mouse scan. Top: Sagittal slice showing the brain volume. Bottom: coronal slice showing the left ventricle (LV), liver and myocardium volumes

Volumes of interest (VOI) were delineated using PMOD 4.0 (PMOD Technologies Ltd., Zurich, Swisserland) in either dynamic or static PET space, the latter which was formed by averaging the last 20 minutes of the dynamic PET acquisition. For the $\mathrm {[^{18}F]FDG}$ data, delineations of myocardium, left ventricle, liver and brain were performed as described in [9]. Briefly, myocardium was delineated as voxels above 40–60% of the maximum voxel value above background in the whole heart in static PET space; Left ventricle was defined as the region encompassed by the myocardium uptake; Brain was delineated as voxels above 50% of the maximum voxel value above background in a region encompassing the entire brain in static PET space (Fig. 2). For $\mathrm {[^{68}Ga]}$-prostate-specific membrane antigen ($\mathrm {[^{68}Ga]PSMA}$ −617) and $\mathrm {[^{18}F]}$-fluorodopa ($\mathrm {[^{18}F]FDOPA}$), the non-specific background uptake in brain and myocardium was significantly lower compared to $\mathrm {[^{18}F]FDG}$. For these tracers, the brain and myocardium regions were, therefore, delineated based on an ellipsoid contained within the skull, and encompassing the myocardium, respectively, based on the coregistered CT images. All volumes of interest were applied to the dynamic PET images, and the mean time-activity curve was extracted from each VOI.

### IDIF calculation

An IDIF was formed for all $\mathrm {[^{18}F]FDG}$ mice by combining the signal from partial-volume corrected left-ventricle data for early time frames, with a blood sample-calibrated liver data for late time frames (Supplementary Figure S6b) [24, 25]. Briefly, the left ventricle time-activity curve was corrected for partial volume effect using Equation 2 in [25]. In this context, the recovery coefficient for the PET/CT and PET/magnetic resonance (MR) systems were measured to 0.42 and 0.44, respectively, for a 1.6 mm diameter left-ventricle [24], while the background activity was defined from the surrounding myocardium region. The liver curve was normalized to a late time-frame blood sample before being combined with the left-ventricle curve. The intersection between the left-ventricle and liver curves was objectively defined for each mouse from the maximum of the second derivative of the left-ventricle signal. Because of the low specific myocardial uptake of $\mathrm {[^{68}Ga]PSMA}$ −617 and $\mathrm {[^{18}F]FDOPA}$, it was not possible to delineate the left ventricle in the mice scanned with these tracers, and consequently, the IDIF was only calculated for the $\mathrm {[^{18}F]FDG}$ mice.

### Input function features

To allow statistical comparison of the AIF and IDIF curves between different groups of mice, six objective curve features were computed from each measured AIF and IDIF (Table 2). The break point between the peak and tail regions was defined objectively as follows: First, the minimum point of the first derivative of the input function was identified, corresponding to the maximum slope. Next, the second positive peak of the second derivative was identified, corresponding to the inflection point where the input function changes concavity. The difference between these two points was calculated and the break point was defined as the inflection point plus half of this difference (Supplementary Figure S6c-d).

**Table 2:** Objective features computed from the AIF and IDIF curves

Feature	Unit	Definition
SUV$_{\text {max}}$	[g/ml]	Maximum value of the input function
TTP	[s]	Time point of the maximum value after injection
FWHM	[s]	Width of the peak at half of its maximum value
AUC$_{\text {peak}}$	[g/ml$\cdot$s]	Area under the peak region
AUC$_{\text {tail}}$	[g/ml$\cdot$s]	Area under the tail region
AUC$_{\text {peak}}$/AUC$_{\text {tail}}$	[1/1]	Ratio of the peak and tail areas

AUC: area under curve, FWHM: full-width at half maximum, SUV: standardized uptake value, TTP: time to peak

### Tracer kinetic modeling

A reversible two-tissue compartment model [1] was fitted to brain and myocardium PET data using both the AIF the IDIF as input function, to obtain rate constants ($K_1$, $k_2$, $k_3$, $k_4$), the fractional blood volume (vB) and the net influx rate constant ($K_\text {i}$) for each tissue. Similarly, Patlak modeling [26] was performed to compute the influx using rate constant ($K_{\text {i,Patlak}}$) for each tissue, using both input functions. Prior to kinetic modeling, dispersion correction was applied to the AIF, and subsequently, both AIF and IDIF were converted to plasma input functions (Supplementary Figure S6). Tracer kinetic modeling was implemented in pyPET, an in-house developed library for tracer kinetic modeling (Python 3.11.5), available at https://github.com/Kuttner/pyPET. The pyPET library was validated against a commercially available kinetic modeling software package prior to use (Supplementary Figures S9 and S10).

### Statistical calculations

The input function features and kinetic modeling parameters were compared between different groups of mice using mean values and 95% confidence intervals. Statistical significance was assessed using the non-parametric Mann-Whitney U-test ($\alpha = 0.05$). All statistical analyses were implemented in Python (v. 3.11.5) using well-documented numerical and statistical libraries (NumPy v. 1.26.4 and SciPy v. 1.15.3, https://docs.scipy.org/doc/). All numerical values are reported as mean ± 95 % confidence interval.

### Triple injection repeatability

In order to evaluate the intra-subject repeatability of the AIF, six mouse experiments were performed with three repeated injections of 30 $\mathrm {\mu l}$ volume of $\mathrm {[^{18}F]FDG}$ with continuous arterial blood sampling from the same arterial cannula. One single blood sample was taken 10 minutes after each injection to allow calibration of the measured AIF curve. The small injection volume of three 30 $\mathrm {\mu l}$ boluses was within the recommended maximum volume for intravenous injections in mice of 5 ml/kg [22]. To adjust for the bias in the second and third AIF measurements caused by circulating radioactivity from prior injections, corrections were applied. First, the second AIF curve was adjusted by subtracting the overlapping portion of the parametric fit from the first AIF curve. Similarly, the third AIF curve was corrected by subtracting the overlapping portions of the parametric fits from both the first and second AIF curves. Finally, the two corrected AIF curves were aligned to the injection time of the first injection. Input function features were calculated from each corrected AIF curve and compared using the previously described statistical methods.

## Results

The results demonstrate that specific experimental conditions led to significant variations in certain AIF and IDIF curve features or kinetic modeling parameters. Table 3 summarizes the key findings for each variable, with detailed results discussed in the following sections.

**Table 3:** Summary of the results. Boldface indicates reference settings

Variable	Value	Results
Injection volume $\mathrm {[\mu l]}$	30 **100** 150	Non-significant differences in curve features 30 $\mu$ l IDIF: Lower AUC ratio 30 $\mu$ l AIF and IDIF: Higher $k_2$ for brain
Injection time $\mathrm {[s]}$	15 **30** 60	15 s AIF and IDIF: Higher peak, shorter TTP 60 s AIF and IDIF: Lower peak, longer TTP, wider FWHM 15 s AIF: Lower $k_2$ for brain 60 s IDIF: Higher $k_2$ for brain
Withdrawal rate $\mathrm {[\mu l/min]}$	30 60 **120**	30 $\mu$ l/min AIF: Lower peak and wider FWHM. 60 $\mu$ l/min AIF: Higher $k_4$ for myocardium. 30 $\mu$ l/min IDIF: Lower $K_1$ for myocardium 60 $\mu$ l/min IDIF: Lower $K_1$ for brain
Age $\mathrm {[weeks]}$	**9** 12 16 20 24	Non-significant differences in curve features 16 weeks: Higher $k_4$ for myocardium (AIF). Lower $K_1$ forbrain (IDIF) 12 weeks: Lower $k_2$ for brain and $K_1$ for myocardium (IDIF) 24 weeks: Lower $k_2$ for myocardium (IDIF)
Strain	**Balb/cJRj** Balb/cJ Balb/cAnNCrl C57BL/6JRj	Balb/cJ AIF: Higher AUC peak and tail C57BL/6JRj AIF: Lower peak, AUC peak, and AUC ratio Balb/cJ AIF: Significant differences in $k_3$, vB, $K_\text {i}$, and $K_{\text {i,Patlak}}$ for brain Balb/cAnNCrl AIF: Significant differences in vB, $K_\text {i}$, and $K_{\text {i,Patlak}}$ for myocardium Similar but not identical kinetic modeling results with IDIF
Radiopharmaceutical	**[**$\mathbf {^{18}F}$**]**$\mathbf{FDG}$ $\mathrm {[^{18}F]FDOPA}$ $\mathrm {[^{68}Ga]PSMA}$ −617	$\mathrm {[^{18}F]FDOPA}$ AIF: Wider FWHM, lower AUC tail, higher AUC ratio $\mathrm {[^{18}F]FDOPA}$ and $\mathrm {[^{68}Ga]PSMA}$ −617: Significant differences in rate constants
PET scanner	**PET/CT** PET/MR	PET/MR AIF and IDIF: Lower peak. PET/MR IDIF: Lower $k_2$ and $k_3$ for brain.
Triple injection	**First injection** Second injection Third injection	Non-significant differences in curve features

AIF: arterial input function, AUC: area under curve, FWHM: full-width at half maximum, IDIF: image derived input function, TTP: time to peak

### Animal experiments

The mouse age across all experiments was 12.2 ± 0.7 weeks, and the corresponding weight was 22.1 ± 0.4 g (Supplementary Figure S3). Fasting time was 3.5 ± 0.1 hours for the $\mathrm {[^{18}F]FDG}$ experiments, with a resulting blood glucose of 6.4 ± 0.3 mmol/l (Supplementary Figure S4). Mice were injected with 15.4 ± 0.7 MBq of radiotracer, and the arterial blood withdrawal rate was 99.0 ± 4.4 $\mathrm {\mu l/min}$ (Supplementary Figure S5). The delay time between the radiation detector and the Y-connector was measured to 30.4 ± 3.4 s.

### Injection volume

**Fig. 3:**
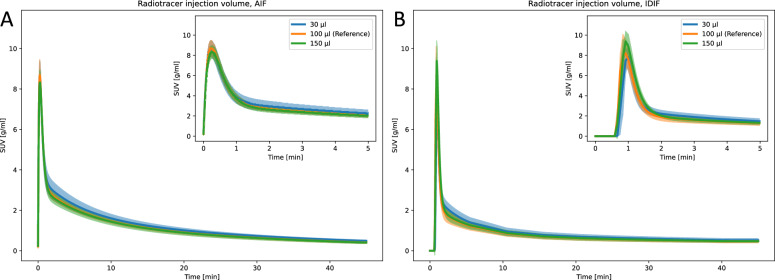
Parametric fits of the (**A**) AIF and (**B**) IDIF curves for varying radiotracer injection volumes. The line and the shaded area represent the mean curve and the 95% confidence interval, respectively

AIF and IDIF curves and kinetic modeling parameters showed no significant differences across injection volumes (Fig. 3 and Supplementary Table S1 and S2). Exceptions were observed in the AUC ratio for IDIF, which was significantly lower for 30 $\mathrm {\mu l}$ (0.093 ± 0.008), compared to 100 $\mathrm {\mu l}$ (0.127 ± 0.022), and in $k_2$ for brain obtained with AIF and IDIF, which was significantly higher for 30 $\mathrm {\mu l}$ (AIF: 0.511 ± 0.055 1/min, IDIF: 0.431 ± 0.034 1/min), compared to 100 $\mathrm {\mu l}$ (AIF: 0.38 ± 0.05 1/min, IDIF: 0.38 ± 0.05 1/min).

### Injection time

**Fig. 4:**
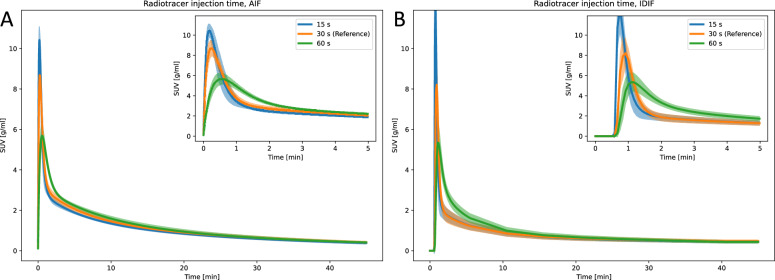
Parametric fits of the (**A**) AIF and (**B**) IDIF curves for varying radiotracer injection times. The line and the shaded area represent the mean curve and the 95% confidence interval, respectively

Shorter injection times (15 s) increased AIF and IDIF peak and decreased time to peak values compared to reference (30 s) injections, while longer times (60 s) had the opposite effect, including a wider FWHM (Fig. 4 and Supplementary Table S3 and S4). Despite differences in curve shapes, the tracer kinetic modeling parameters showed non-significant differences across injection times for both AIF and IDIF. Exception were observed in $k_2$ calculated with AIF for brain, which was significantly lower for 60 s injection time (0.377 ± 0.022 1/min), compared to 30 s injection time (0.431 ± 0.034 1/min), and in $k_2$ calculated with IDIF for brain, which was significantly higher for 60 s (0.482 ± 0.059 1/min), compared to 30 s (0.38 ± 0.05 1/min) (Supplementary Tables S3 and S4).

### Withdrawal rate

**Fig. 5:**
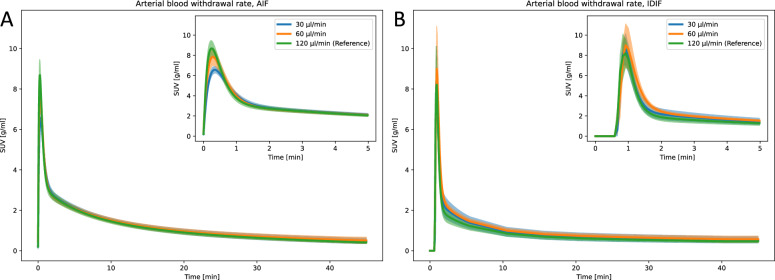
Parametric fits of the (**A**) AIF and (**B**) IDIF curves for varying arterial blood withdrawal rates. The line and the shaded area represent the mean curve and the 95% confidence interval, respectively

Lowering the withdrawal rate (30$\mathrm {\mu l/min}$ and 60 $\mathrm {\mu l/min}$) resulted in lower and wider AIF peak compared to reference (120 $\mathrm {\mu l/min}$) (30 $\mathrm {\mu l/min}$: all P < 0.05, 60 $\mathrm {\mu l/min}$: n.s.), while there were no significant differences among the IDIF curve features (Fig. 5 and Supplementary Tables S5 and S6). Despite the different AIF curve shapes, the tracer kinetic modeling parameters using both AIF and IDIF were similar to, and non-significantly different from reference, for both 60 $\mathrm {\mu l/min}$ and 30 $\mathrm {\mu l/min}$ withdrawal rates. Exception were observed in AIF $k_4$ for brain, which was significantly higher for 60 $\mathrm {\mu l/min}$ (0.018 ± 0.007) compared to 120 $\mathrm {\mu l/min}$ (0.04 ± 0.005) (Supplementary Table S5), in IDIF $K_1$ for brain, which was significantly lower for 60 $\mathrm {\mu l/min}$ (0.187 ± 0.027 ml/min/ml), compared to 120 $\mathrm {\mu l/min}$ (0.237 ± 0.024 ml/min/ml), and in IDIF $K_1$ for myocardium, which was significantly lower for 30 $\mathrm {\mu l/min}$ (0.6 ± 0.129 ml/min/ml), compared to 120 $\mathrm {\mu l/min}$ (1.14 ± 0.398 ml/min/ml) (Supplementary Table S6).

### Mouse age

**Fig. 6:**
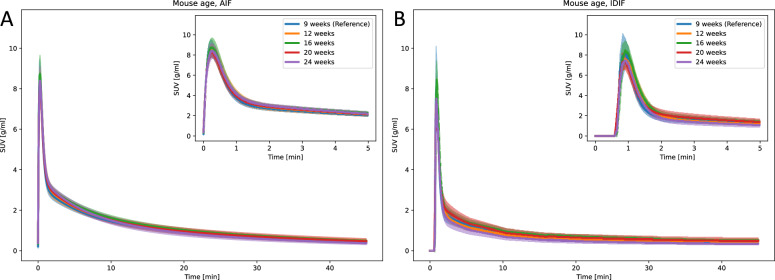
Parametric fits of the (**A**) AIF and (**B**) IDIF curves for varying mouse ages. The line and the shaded area represent the mean curve and the 95% confidence interval, respectively

AIF and IDIF curves and most kinetic modeling parameters showed non-significant differences across mouse ages (Fig. 6, Supplementary Table S7 and S8). Exceptions were observed in $k_4$ for myocardium obtained with AIF, which was significantly higher for 16-week-old mice (0.016 ± 0.05 1/min), compared to 9-week-old mice (0.04 ± 0.005 1/min). Some kinetic parameters obtained using IDIF also showed significant differences from the reference group. For 16-week-old mice, $K_1$ in the brain was lower (0.178 ± 0.009 ml/min/ml) compared to the reference value (0.237 ± 0.024 ml/min/ml). Similarly, for 12-week-old mice, $k_2$ in the brain was lower (0.3 ± 0.071 1/min) compared to the reference value (0.38 ± 0.05 1/min). In the myocardium, $K_1$ was also lower for 12-week-old mice (0.646 ± 0.287 ml/min/ml) and compared to the reference value (1.14 ± 0.398 ml/min/ml), while $k_2$ for 24-week-old mice was lower (0.544 ± 0.945 1/min) compared to reference (2.625 ± 2.502 1/min).

### Mouse strain

**Fig. 7:**
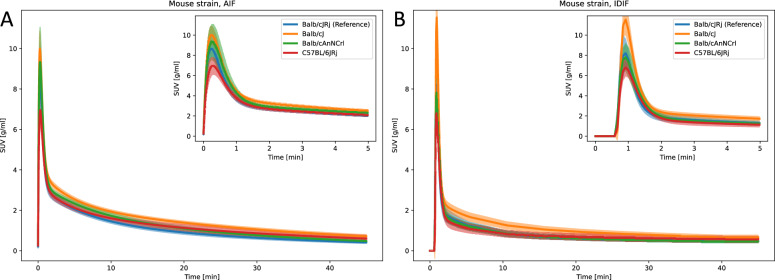
Parametric fits of the (**A**) AIF and (**B**) IDIF curves for varying mouse strains. The line and the shaded area represent the mean curve and the 95% confidence interval, respectively

The AIF peak, time to peak, and FWHM values for Balb/cJ and Balb/cAnNCrl mice were similar to the reference Balb/cJRj strain, while C57BL/6JRj mice showed a significantly lower peak (6.98 ± 0.869 g/ml) compared to Balb/cJRj (8.781 ± 0.747 g/ml) (Fig. 7a and Supplementary Table S9). Balb/cJ mice exhibited a slightly higher AUC peak and tail compared to reference Balb/cJRj mice, which was not observed for the Balb/cAnNCrl strain. C57BL/6JRj mice also had a lower AIF AUC peak compared to Balb/cJRj. Kinetic modeling parameters were significantly different for Balb/cJ and Balb/cAnNCrl strains, compared to the reference strain, for instance $k_3$, $K_i$, and $K_{\text {i,Patlak}}$ for Balb/cJ mice and vB, $K_i$, and $K_{\text {i,Patlak}}$ for myocardium for Balb/cAnNCrl (Supplementary Table S9). Similar results were obtained from the IDIF measurements (Fig. 7b and Supplementary Table S10), but with notable differences in, for instance, the FWHM for Balb/cAnNCrl, which was significantly wider (35.015 ± 3.779 s) compared to the reference strain (28.123 ± 3.451 s). Unlike AIF, IDIF-based kinetic modeling for Balb/cAnNCrl did not show significant differences in myocardium tissue compared to the reference strain.

### Radiopharmaceutical

**Fig. 8:**
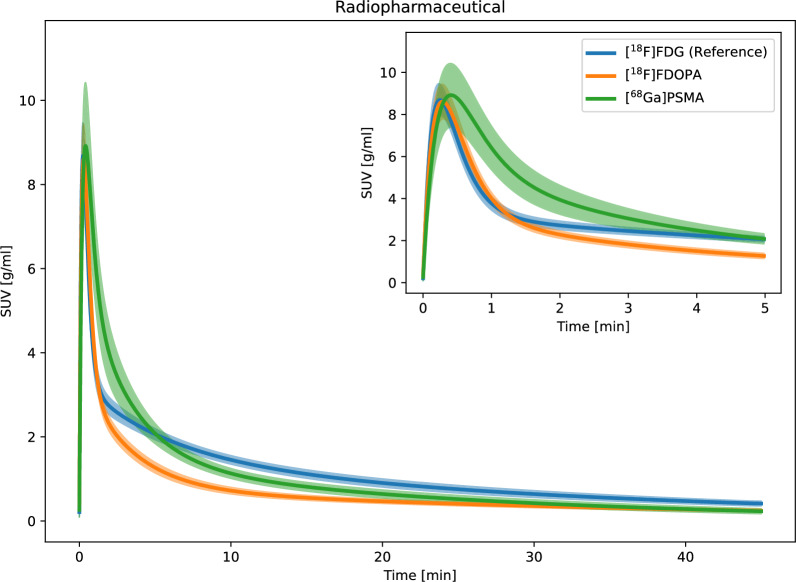
Parametric fits of the AIF curves for varying injected radiopharmaceuticals. The line and the shaded area represent the mean curve and the 95% confidence interval, respectively

The AIF FWHM for $\mathrm {[^{18}F]FDOPA}$ was significantly wider than for $\mathrm {[^{18}F]FDG}$ ($\mathrm {[^{18}F]FDOPA}$: 39.683 ± 1.829 s, $\mathrm {[^{18}F]FDG}$: 35.237 ± 3.078 s), with a lower AUC tail ($\mathrm {[^{18}F]FDOPA}$: 26.61 ± 2.562 g/ml $\cdot$ min, $\mathrm {[^{18}F]FDG}$: 43.468 ± 3.298 g/ml $\cdot$ min), and a higher AUC ratio ($\mathrm {[^{18}F]FDOPA}$: 0.176 ± 0.017, $\mathrm {[^{18}F]FDG}$: 0.104 ± 0.01) (Fig. 8a and Supplementary Table S11). In contrast, the AIF for $\mathrm {[^{68}Ga]PSMA}$ −617 was similar to $\mathrm {[^{18}F]FDG}$, with no significant differences in curve features. As expected, most kinetic modeling rate constants for brain and myocardium tissues were significantly different for both $\mathrm {[^{18}F]FDOPA}$ and $\mathrm {[^{68}Ga]PSMA}$ −617 compared to $\mathrm {[^{18}F]FDG}$ values (Fig. 8b and Supplementary Table S11).

### PET scanner

**Fig. 9:**
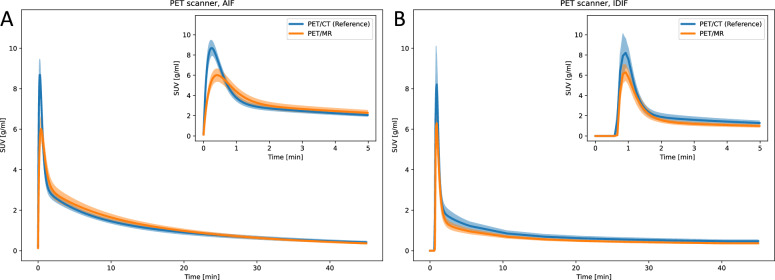
Parametric fits of the (**A**) AIF and (**B**) IDIF curves for varying PET scanners. The line and the shaded area represent the mean curve and the 95% confidence interval, respectively

The AIF and IDIF peaks were significantly lower in PET/MR (AIF: 6.024 ± 0.620 g/ml, IDIF: 6.285 ± 0.864 g/ml), compared to PET/CT (AIF: 8.781 ± 0.747 g/ml, IDIF: 8.969 ± 1.356 g/ml) (Fig. 9a, and Supplementary Table S12). AIF-based kinetic modeling parameters were similar between scanners, but IDIF-based kinetic modeling showed significantly lower $k_2$ and $k_3$ for brain in PET/MR compared to PET/CT (Figure 9b and Supplementary Table S13).

### Triple injection repeatability

**Fig. 10:**
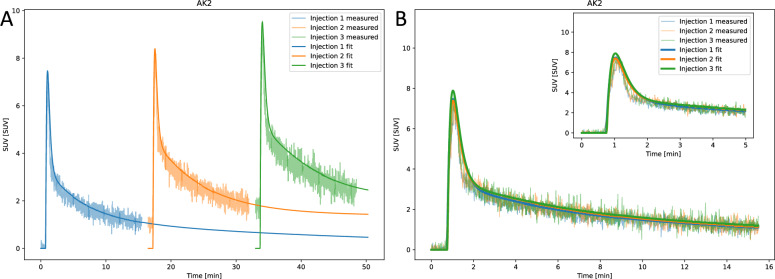
Representative example from the triple injection repeatability experiments. (**A**) Uncorrected AIF measurements with corresponding parametric fits for each of the three consecutive injections of $\mathrm {[^{18}F]FDG}$ in the same mouse during the same arterial cannulation experiment. (**B**) The second AIF curve was corrected by subtracting the overlapping part of the parametric fit from the first AIF curve. The third AIF curve was corrected by subtracting the overlapping part of the parametric fit from the first and second AIF curves. Finally, the two latter corrected AIF curves are shifted to the injection time of the first injection

After correcting for overlap and delay, the three AIF curves aligned closely (Fig. 10). Comparison across the six experiments revealed only minor, non-significant differences between the first reference injection and the subsequent two injections (Supplementary Table S14).

## Discussion

The availability of datasets for training deep learning models to predict the AIF in small-animal PET imaging has been limited to small sample size, specific tracers, and reliance on image-derived labels [9, 11]. Understanding the variability of input data and training labels under different experimental conditions is critical for assessing applicability and limitations DLIF models. For instance, it has been shown that a poor estimation of the input function shape, by using a population-based input function in a $\mathrm {[^{18}F]FDG}$ study of mice, resulted in individual errors greater than 10% in the estimation of cerebral metabolic rate of glucose, compared to arterial blood sampling [27]. Therefore, in this prospective study, we acquired a comprehensive dataset of dynamic PET data with AIF measurements from 112 mice under controlled and varied experimental conditions. The dataset allowed us to investigate the AIF and IDIF variability and to calculate reference kinetic modeling parameters across different experimental settings. The key findings, summarized in Table 3, showed that mouse strain, injection time, withdrawal rate, and PET scanner (tubing length) significantly affected the shapes of the AIF and IDIF peaks, while injection volume and mouse age did not introduce bias. Additionally, different radiopharmaceuticals resulted in distinct differences in AIF curves. Each variable is discussed in detail in the following subsections.

### Injection volume

Injection volume can affect animal physiology and welfare, for why guidelines recommend maximum allowed injection volume of 5 ml/kg into rodents [22, 28]. In this study, the reference group recieved a 100 $\mathrm {\mu l}$ $\mathrm {[^{18}F]FDG}$ injection, corresponding to the maximum allowed intravenous bolus volume for a 20 g mouse. To enable triple injection repeatability experiments, we first investigated whether multiple small-volume injections (30 $\mathrm {\mu l}$) could be performed without altering the AIF and IDIF shapes. Additionally, we assessed the impact of larger injection volumes (150 $\mathrm {\mu l}$) on these input functions. Both the AIF and the IDIF curves were visually and numerically similar across all groups (Fig. 3, Supplementary Table S1-S2). These findings suggest that injection volume variability does not bias a DLIF model trained with a specific injection volume. Furthermore, they confirm the feasibility of triple injection experiments with three 30 $\mathrm {\mu l}$ boluses and indicate that small volume variations during manual radiotracer preparation do not affect the measured input functions.

### Injection time

Intravenous bolus injection into the tail vein is a common method for administering radiopharmaceuticals in dynamic PET imaging studies of mice [18]. Current guidelines recommend short injection times of approximately 1 min [22]. To investigate the impact of injection time on the measured AIF and IDIF, mice were injected during 15 s, 30 s (reference) and 60 s (Table 1). As expected, both visual (Fig. 4) and quantitative analyses (Supplementary Tables S3 and S4) showed significant changes in AIF and IDIF peak shapes with varying injection times, with shorter injections resulting in higher peak amplitudes. However, the AUC peak and tail remained constant, as the total injection volume was unchanged. The consistent AUC values between AIF and IDIF suggest that a DLIF model, which relies on image-based features, would likely be invariant to injection time. Additionally, most kinetic modeling parameters remained stable across injection times. This is consistent with the model equations, which incorporate the area under curve through convolution of the input function with an exponential factor [29]. Consequently, when the AUC peak and tail are preserved, the kinetic modeling parameters remain unchanged.

### Withdrawal rate

The arterial blood withdrawal rate is critical for accurate sampling of the input function peak. We found that lower withdrawal rates resulted in a lower and wider AIF peak, while the AIF tail remained unchanged (Fig. 5, Supplementary Table S5 and S6). This is coherent with previous studies, attributing the lower measured AIF peak at lower withdrawal rates with increased dispersion effect [14, 15, 30]. As expected, the IDIF peak was unaffected, as it is derived entirely from image data. Interestingly, despite the changes in AIF peak amplitude and width, kinetic modeling parameters remained stable across different withdrawal rates. This can be explained by the consistent AUC across withdrawal rates, which, as previously discussed, does not affect kinetic modeling. While a DLIF model should ideally be trained with AIF labels sampled at the highest possible withdrawal rate to minimize dispersion effects, our findings indicate that lower withdrawal rates still yield consistent kinetic modeling results for the data analyzed in this study.

### Mouse age

The AIF and IDIF curves from mice of different ages (9 to 24 weeks) were visually similar (Fig. 6), and statistical analysis showed non-significant differences between the age groups (Supplementary Table S7 and S8). Only a few kinetic modeling parameters differed significantly across age groups, likely reflecting physiological changes with age. For example, Brendel et al. reported age-dependent cortical hypermetabolism of glucose in 56-week-old wild-type mice compared to 20-week-old animals [31], while Zhao et al observed increased $\mathrm {[^{18}F]FDG}$ uptake in all organs of 96-week-old mice compared to 9-week-old mice [32]. Our findings suggest that mice within the tested age range can be safely included in training a DLIF model. Consequently, a trained DLIF model would likely generalize well to mice within this age range, but this has to be validated in future studies.

### Mouse strain

Mouse strain can significantly influence PET uptake values due to physiology differences. For example, Berglund et al. reported variations in whole-body glucose metabolism across inbred mouse strains [16], and Shah et al. observed strain-dependent differences in $\mathrm {[^{18}F]FDG}$ uptake in several brain regions, including comparisons between Balb/C and C57BL/6 mice [33]. Therefore, it is important to assess whether a DLIF model trained on one strain can generalize to others by evaluating potential differences in AIF and IDIF measurements. In this study, the peak, time to peak, and FWHM were consistent in all Balb/c strains, while C57Bl/6JRj mice exhibited significantly lower AIF and IDIF peak and AUC values compared to reference Balb/cJRj mice (Fig. 9 and Supplementary Tables S9 and S10). These differences may reflect strain-specific variations in tracer clearance from the blood and could indicate that certain strains require individually trained DLIF models, which should be investigated in future studies. Interestingly, despite these differences, kinetic modeling parameters for C57Bl/6JRj mice were not significantly different from the reference strain, possibly due to corresponding differences in the measured tissue curves. Balb/cJ mice, bought from a different supplier (The Jackson Laboratory, Ellsworth, Maine, US) than the reference strain (Janvier, Le Genest-Saint-Isle, France), showed higher AUC values and significantly lower brain kinetic parameters compared to the reference strain (Fig. 7 and Supplementary Tables S9 and S10), suggesting strain-dependent differences. Our findings for C57BL/6JRj mice align with published $K_\text {i, Patlak}$ data for brain tissue, but the observed myocardium influx rates were higher than previously reported [18, 19]. This discrepancy is likely due to differences in fasting duration, as mice in these these studies were fasted overnight, while our mice fasted for 3.5 h. Longer fasting times are known to reduce tracer influx in the myocardium [18, 19].

### Radiopharmaceutical

The radiopharmaceuticals evaluated in this study exhibit different biological uptake and clearance patterns. While $\mathrm {[^{18}F]FDG}$ is a glucose analogue to asses glucose metabolism [34], $\mathrm {[^{18}F]FDOPA}$ is a dopamine precursor used to assess brain dopamine synthesis and amino acid transport [35]. Meanwhile, $\mathrm {[^{68}Ga]PSMA}$ −617 is a prostate-specific membrane antigen-targeting agent originally developed for imaging prostate cancer. Visual comparison revealed distinct AIF curves for the three radiopharmaceuticals, and statistical analysis identified significant differences in curve features between $\mathrm {[^{18}F]FDG}$ and $\mathrm {[^{18}F]FDOPA}$, while $\mathrm {[^{68}Ga]PSMA}$ −617 was similar to the reference (Supplementary Table S11). However, nearly all kinetic modeling parameters differed for $\mathrm {[^{18}F]FDOPA}$ and $\mathrm {[^{68}Ga]PSMA}$ −617, compared to $\mathrm {[^{18}F]FDG}$, reflecting differences in tissue uptake among the radiopharmaceuticals. These findings suggest that a DLIF model trained on $\mathrm {[^{18}F]FDG}$ data may have limited applicability to radiopharmaceuticals with vastly different uptake patterns, such as $\mathrm {[^{18}F]FDOPA}$ or $\mathrm {[^{68}Ga]PSMA}$ −617, which we have also shown in a preliminary study [12].

### PET scanner

The impact of the PET scanner should, ideally, not affect the measured AIF or IDIF, as this could introduce bias in multicenter studies. However, the two PET imaging systems at the UiT facility had significantly different geometries. The PET/CT system allowed direct access to the mouse with short injection and withdrawal catheters, while the PET/MR system required longer tubing, with the mouse positioned inside the MR bore (Supplementary Figure S1). It is well-established that longer tubing increases tracer dispersion in human PET imaging, as shown in studies using tubing lengths of 150–450 cm with a 1 mm inner diameter [36]. Our results confirm similar dispersion effect in small-animal PET, where PE10 tubing with a 0.28 mm inner diameter was used. This is evident from the significantly lower AIF peak measured with the PET/MR scanner compared to the PET/CT system (Fig. 9 and Supplementary Tables S12 and S13). Additionally, the longer tubing in the PET/MR caused a measurable decrease in withdrawal rate (Supplementary Section S17 and Supplementary Figure S7). Applying dispersion correction to the PET/MR data partially restored the AIF peak amplitude, bringing it closer to that of the PET/CT measurements (Supplementary Section S18 and Supplementary Figure S8). This highlights the importance of dispersion correction, especially when long tubing is used. We speculate that the DLIF model could, in principle, be trained using either dispersed or corrected AIF data as labels, as the model in the latter case would likely embed the dispersion correction into its parameters. To reduce complexity, our current DLIF approaches are based on dispersed AIF data [11, 12], as dispersion correction can be performed independently and after DLIF model prediction.

### Triple injection repeatability

Multi-tracer injections in mice are challenging due to overlapping signals and noise in the measured data [37]. While previous studies have demonstrated dual or triple injections, these were often performed with different tracers, without simultaneous AIF measurement for kinetic modeling [37, 38], or with at least one hour between injections [39]. In this study, we tested the repeatability of AIF measurements in six mice using three repeated injections of 30 $\mu$l $\mathrm {[^{18}F]FDG}$, administered 15 minutes apart. After necessary corrections for circulating tracer from previous injections, the three measured AIF curves showed strong overlap with no significant differences in curve features. These findings demonstrate that AIF measurements within the same subject are highly repeatable, and it further suggests that the variability between subjects and groups observed in this study is likely due to biological differences or variations in the experimental setup.

### Limitations

This study has several limitations. The small sample size in each experimental group required the use of the non-parametric Mann-Whitney U-test for comparing AIF and IDIF curve features, as normality could not be assumed. The limited sample size may also account for the wide confidence intervals observed in some kinetic modeling parameters. Despite the limited data, a subset of this dataset was used to prospectively train a DLIF model in a preliminary study [12], demonstrating that the dataset provided a sufficient sample size for accurate DLIF model training. Another limitation is that only a three radiopharmaceuticals, based on either $\mathrm {^{18}F}$ or $\mathrm {^{68}Ga}$, were evaluated in this study, as we had limited availability of other radioisotopes in our imaging facility.

## Conclusions

This study collected a comprehensive dataset of dynamic PET data with AIF measurements from 112 mice under controlled experimental conditions to evaluate the variability of AIF, IDIF, and kinetic modeling parameters. We found that factors, such as mouse strain, injection time, withdrawal rate, and PET scanner (tubing length) significantly influenced AIF and IDIF peak shapes, while injection volume and mouse age did not introduce bias. Additionally, the choice of radiopharmaceutical led to distinct differences in AIF curves. These findings provide crucial knowledge for data curation for DLIF model training, and for understanding the applications and limitations of DLIF across diverse experimental conditions.

## Supplementary Information

Supplementary Material 1

## Data Availability

The datasets generated analysed during the current study are not publicly available due to protection of intellectual property and innovation, but are available from the corresponding author on reasonable request.

## Abbreviations

AIF: Arterial input function
AUC: Area under curve
CI: Confidence interval
CT: Computed tomography
DLIF: Deep learning based input function
FDG: Fluorodeoxyglucose
FWHM: Full-width at half maximum
IDIF: Image-derived input function
MR: Magnetic resonance
PET: Positron emission tomography
SUV: Standardized uptake value
TTP: Time to peak
vB: Fractional blood volume
VOI: Volume of interest
