# Supplementary material for: Variability of the arterial input function in small-animal dynamic PET imaging
Source: EJNMMI Res. 2026 Feb 11;16:42. doi: 10.1186/s13550-025-01367-8 (PMC12996576; doi:10.1186/s13550-025-01367-8)
Supplement: Supplementary file 1 — Supplementary Material 1 [file 13550_2025_1367_MOESM1_ESM.pdf]

Supplementary material

Variability of the arterial input function in small-animal dynamic PET imaging

Samuel Kuttner[1,2,3*], Rodrigo Berzaghi[3], Laurence Convert[4], Otman Sarrhini[4], Roger Lecomte[4,5], Rune Sundset[1,3]

[1]The PET Imaging Center, University Hospital of North Norway, Norway.
[2]UiT Machine Learning Group, Department of Physics and Technology, UiT The Arctic University of Norway, Norway.
[3]Nuclear Medicine and Radiation Biology Research Group, Department of Clinical Medicine, UiT The Arctic University of Norway, Norway.
[4]Sherbrooke Molecular Imaging Centre of CRCHUS and Department of Medical Imaging and Radiation Sciences, Université de Sherbrooke, QC, Canada
[5]Imaging Research & Technology Inc., Sherbrooke, QC, Canada

[*]Corresponding and first author:
Samuel Kuttner
The PET Imaging Center
University Hospital of North Norway
9038 Tromsø Norway
e-mail: samuel.kuttner@uit.no

S1 Animals

In total, 112 healthy female mice were included in this study. BALB/cJRj and C57BL/6JRj mice were purchased from Janvier (Le Genest-Saint-Isle, France), Balb/cAnNCrl mice were bought from Charles River Laboratories (Wilmington, MA, USA), and Balb/cJ mice were purchased from The Jackson Laboratory (Ellsworth, Maine, US). All mice were 7-8 weeks of age when they arrived at the animal facilities at UiT and UdS. They were housed in standard animal housing rooms in groups of 4 in either open euro standard type III or IVC cages. For the age experiments, mice were housed in-house and included in the experiments once they reached the desired age. Animals were fed with a standard rodent diet (UiT: Ssniff V-1554-300 maintenance, Soest, Germany, UdS: Inotiv 2920X Teklad irradiated global soy protein-free extruded, Lafayette, IN, USA). They received water and food ad libitum. The mice that were included in [^{18}F]FDG experiments were fasted for 3 hours before radiotracer injection, while no fasting was performed for the mice injected with the other radiotracers.

S2 Venous and arterial cannulation

Following initial anesthesia with 3-5% isoflurane in 2 l/min oxygen, the mice were weighed and placed on a heated plate (38°C) connected to a vaporizer mask delivering a maintenance dose of 1.5-2% isoflurane in 1-1.5 l/min oxygen. A 15 cm PE10 catheter (BD Intramedic, Mississauga, Canada) was inserted into the tail vein of the animal and secured with a micropore tape. This catheter was used for both radiotracer administration and for the arterial-venous shunt, described later. Blood glucose was measured during venous cannulation using a glucose meter (FreeStyle Lite (UiT) / Freestyle Precision Neo (UdS), Abbott Laboratories, Chicago, USA).

Surgical cannulation of the carotid artery was performed using a 30 cm PE10 with 1 cm PE5 (BD Intramedic, Mississauga, Canada) inserted in one of the extremities. Inspired by a previously published protocol [1], briefly, the mouse neck area was cleaned with ethanol 70%, and an incision was made to the right of the midline of the neck. Fat and muscle were separated to expose the trachea. The carotid artery was located running parallel to the trachea and carefully isolated from vagus nerve. An incision was made in the artery using a surgical micro scissor and the PE5 end of the catheter was carefully inserted into the artery and secured with surgical sutures. The arterial line was run through a radiation detector (UiT: Twilite Two; Swisstrace GMBH, Menzingen, Swisserland; UdS: Ultra High Sensitivity Blood Counter [2]) and a peristaltic pump (UiT: Masterflex Ismatec Microflow; VWR, Radnor, PA, USA, UdS: P625/900.133, Instech Laboratories Inc., Plymouth, Meeting, PA, USA) to measure the whole blood radioactivity concentration with 1 s temporal resolution, simultaneously with the PET scan. Inspired by previously published methodlogy [3], an arterial-venous shunt was created between the carotid artery and the tail vein of the mouse, to allow continuous arterial line measurements during the entire PET scan without excessive blood loss. A 25G, 3-way Y connector (Instech Laboratories Inc., Plymouth Meeting, PA, USA) allowed for intravenous injection of the radiotracer into the closed shunt while circulating blood (Main paper Figure 1). The dead volume of the injection catheter (20 μl) was added to the desired injection volume when preparing the syringe for radiotracer injection. All PE catheters were heparin-coated prior to cannulation.

S3 Manual arterial blood samples

In order to calibrate the continuous line radiation detector, and to measure the actual arterial withdrawal rate during each mouse scan, three manual blood samples were taken in a late stage, or after the PET scan was finished. The arterial end of the PE tube was disconnected from the Y-connector, allowing blood to drip into a pre-weighed eppendorf tube for 30 s. This process was repeated three times, either by reconnecting the PE tube to the Y-connector and allowing at least one minute of arterial venous circulation between samples (UiT), or by withdrawing all samples consecutively without interruption (UdS). The radioactivity concentration of the manual blood samples was measured using a cross-calibrated gamma counter (UiT: 2480 Wizard^2, PerkinElmer, Inc. MA, USA; UdS: Hidex AMG 425-601, Hidex Oy, Turku, Finland). During initial setup of the shunt, as arterial blood passed through the radiation detector during the first pass, the time was measured between the radiation detector and the Y-connector, to allow for delay correction of the manual blood sample measurements and the reading from the radiation detector. This delay time was usually in the order of 20-30 s.

S4 Processing of the continuous arterial blood data to form the AIF

The calibration factor for the continuous line measurement at each manual blood sample time point was obtained as the ratio of the average radioactivity concentration measured in the blood sample to the average signal from continuous radiation detector during the 30 s blood sample time, corrected for delay. If one of the blood sample

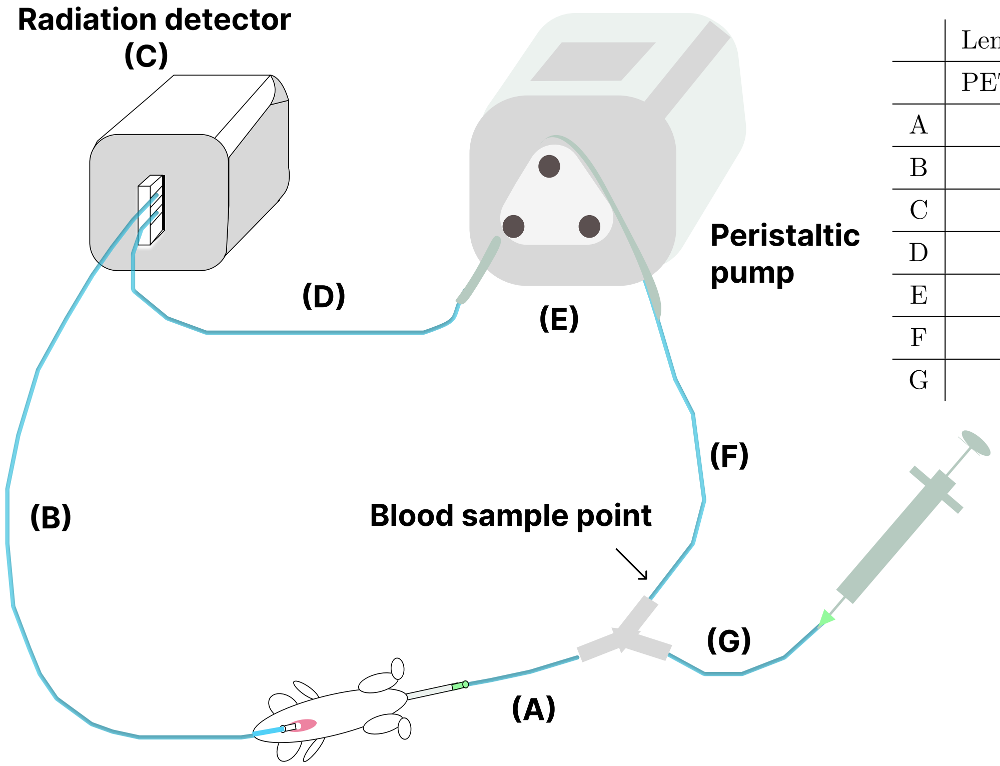

	Length of tubing (cm)	
	PET/CT	PET/MR
A	15	40
B	30	80
C	20	20
D	20	20
E	30	30
F	20	30
G	10	15

Figure S1: Experimental setup of the arterio-venous shunt, tracer injection and continuous arterial line measurements. The table inset shows the tubing lengths for PET/CT and PET/MR experiments, respectively.

calibration factors were outside three scaled median absolute deviations away from the median factor, it was considered as outlier and excluded [4]. The overall calibration factor was obtained as the average factor from the included blood sample factors. The final AIF for each mouse was obtained by multiplication of the continuous line measurement data with the average calibration factor. All following calculations were done on these calibrated AIF curves. In order to enhance the visualization of noisy AIF curves, a well-known parametric model was fit to the calibrated AIF data [5]. One of the model parameters, the timing delay constant, was used for delay correction to allow for comparison of parametric AIF curves across mice and experiments. Figure S2 shows an example of a measured and calibrated AIF, as well as the original and delay corrected parametric fit.

S5 Image acquisition

The PET/CT scans were performed using Triumph^{TM} LabPET-8^{TM} small animal PET/CT scanners (TriFoil Imaging Inc., Chatsworth, CA, USA). PET/MR imaging was performed using a hybrid PET and 7 T magnetic resonance imaging system (MR solutions, Guildford, UK). The anesthetized mice were centered in the field-of-view of the PET scanner (Main paper Figure 1), while lying on a 35^oC heated bed, with a sensor monitoring the breathing rate. While continuously looping blood through the arterial-venous shunt, a 45.5-minute listmode PET acquisition was started. The radiotracer injection (15.4 ± 0.7 MBq) was performed by using an automated injection pump, which started 30 s after PET acquisition start. CT imaging (PET/CT) and a Dixon MR imaging sequence (PET/MR) was performed for PET attenuation and scatter correction. Following PET/CT and PET/MR imaging, while the mice were still in deep anesthesia, they were euthanized using cervical dislocation. To account for variations in PET scanner sensitivity, a phantom (PET/CT: 50 ml syringe, PET/MR: 5 ml syringe) filled with a known concentration (PET/CT: 0.23 ± 0.02 MBq/ml, PET/MR: 1.15 ± 0.37 MBq/ml) of [^{18}F]FDG was scanned daily.

S6 Image reconstruction and processing

The PET images were reconstructed into 42 time frames (1×30, 24×5, 9×20, and 8×300 s) using a 3-dimensional maximum-likelihood estimator algorithm with 50 iterations (PET/CT) or a 3-dimensional ordered subset expectation maximization algorithm with 1 iteration and 32 subsets (PET/MR). Corrections for detector efficiency,

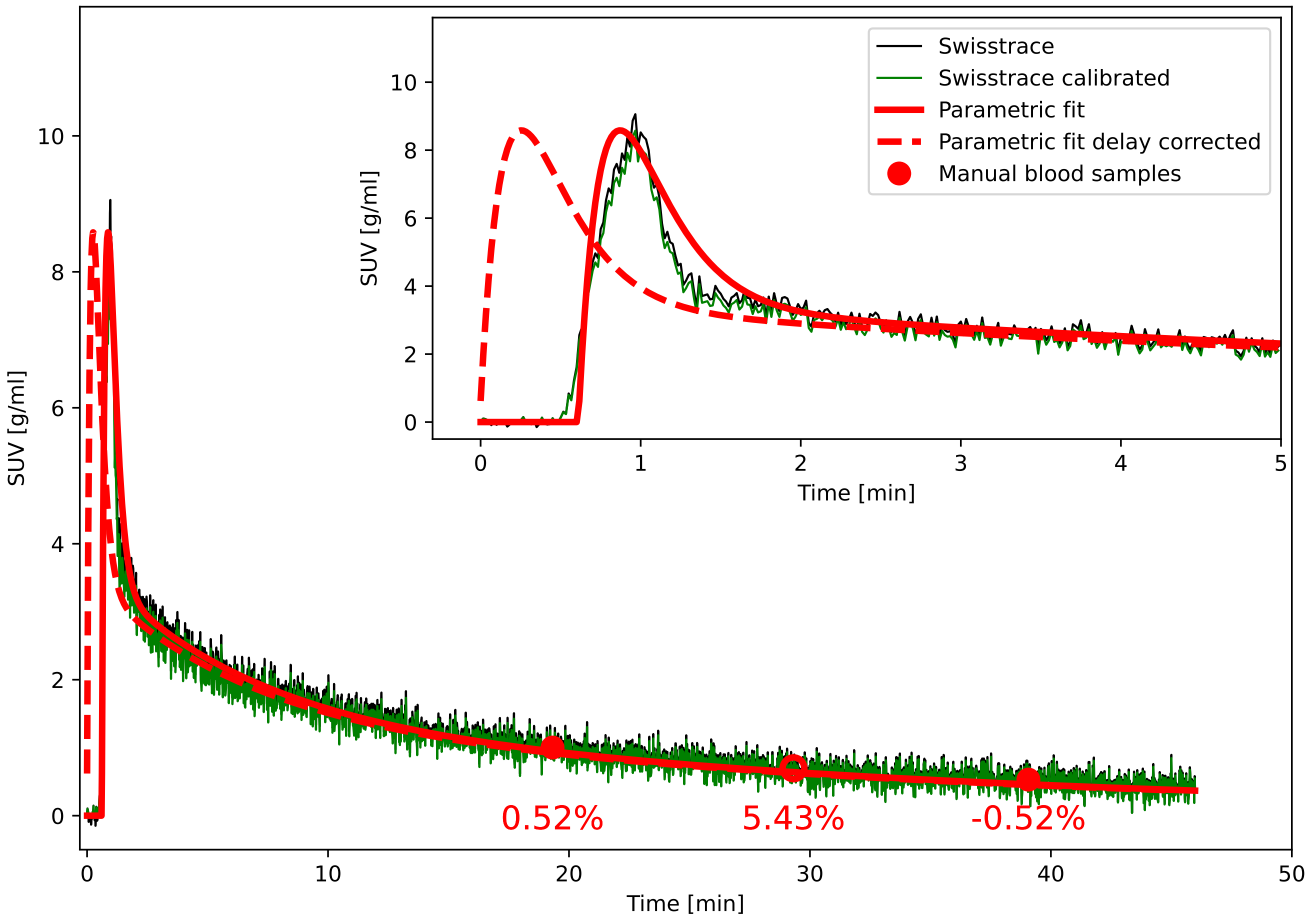

Figure S2: Example of a measured and calibrated AIF, as well as the original and delay corrected parametric fit.

radioactive decay, random coincidences, dead time, attenuation and scatter were applied. The matrix size was 128×92×92 voxels (PET/CT) or 360×86×86 voxels (PET/MR). The voxels were converted into units of MBq/ml using the average counts inside a 14 ml (PET/CT) or 1 ml (PET/MR) cylindrical image region of the daily phantom scan. Subsequently, the voxels were normalized into standardized uptake value (SUV) [g/ml].

S7 Demography and experimental variables

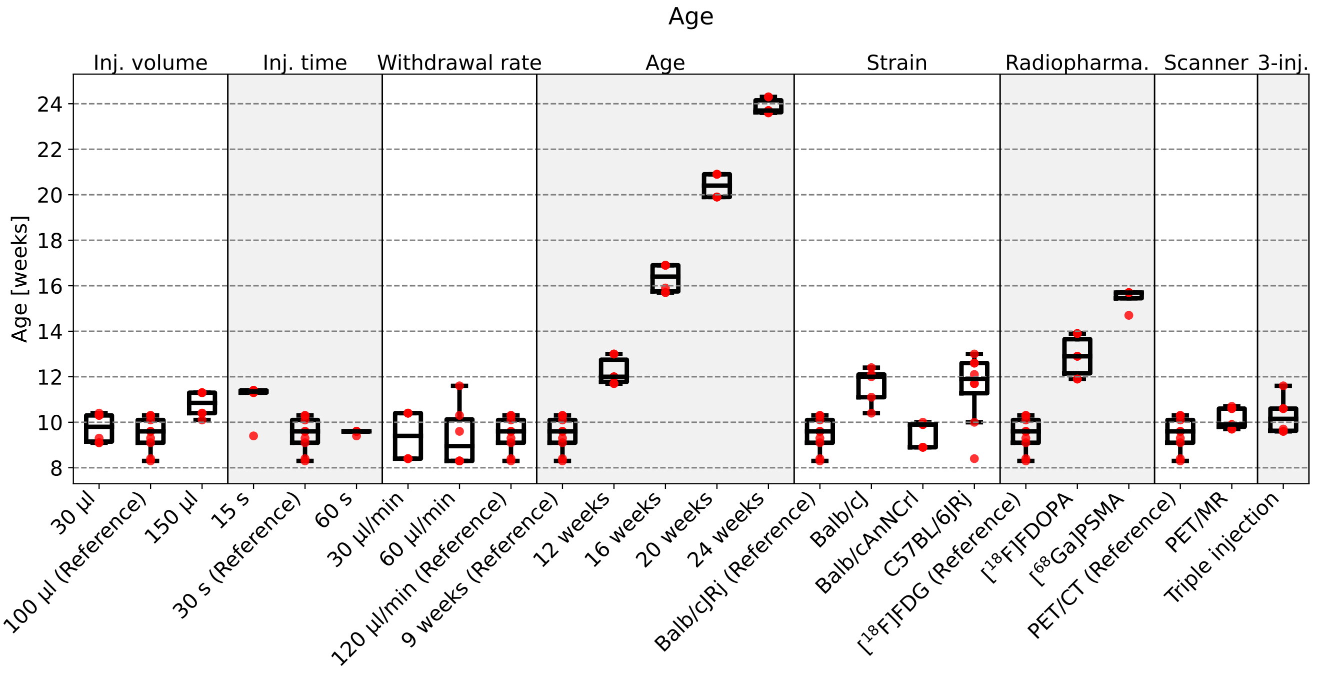

(a)

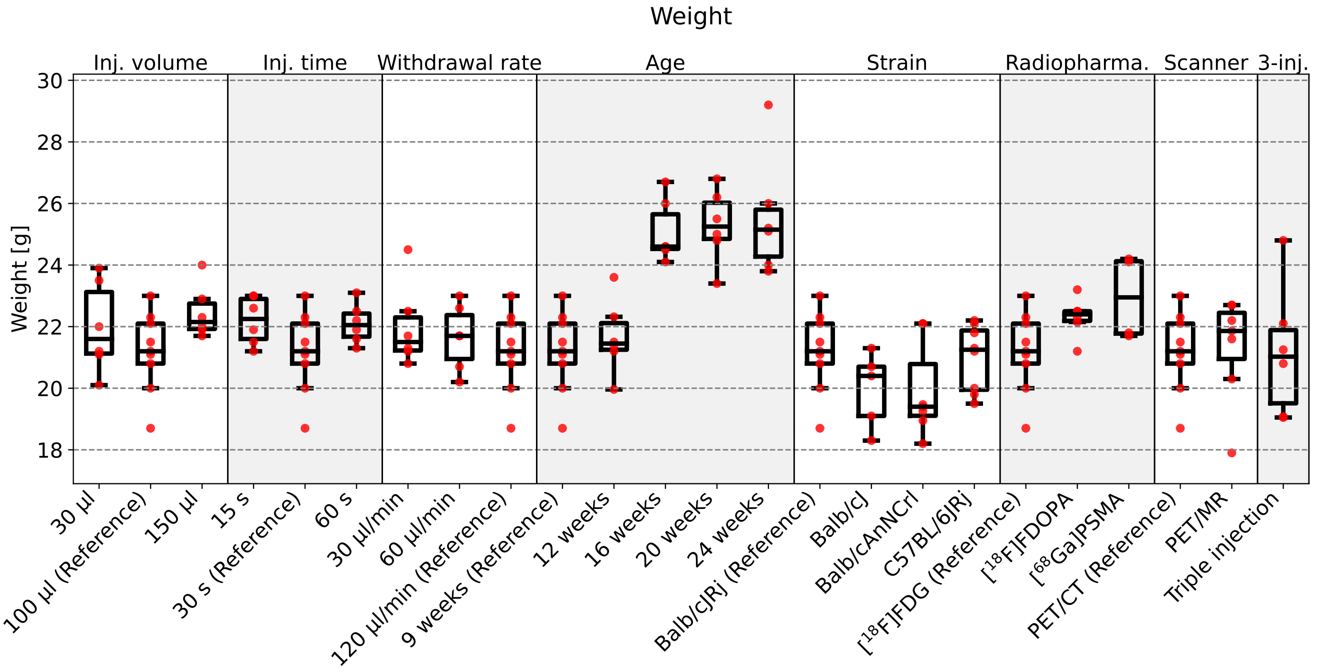

(b)

Figure S3: Distribution of age (a) and weight (b) of the mice for each experiment. Note that the reference distribution in each experiment correspond to the same data. In the boxplot, the horizontal line and the black box represent median and interquartile range (25th to 75th percentile), respectively, while the whiskers indicate the maximum and minimum data point up to 1.5 × interquartile range. Individual data points are shown with red dots.

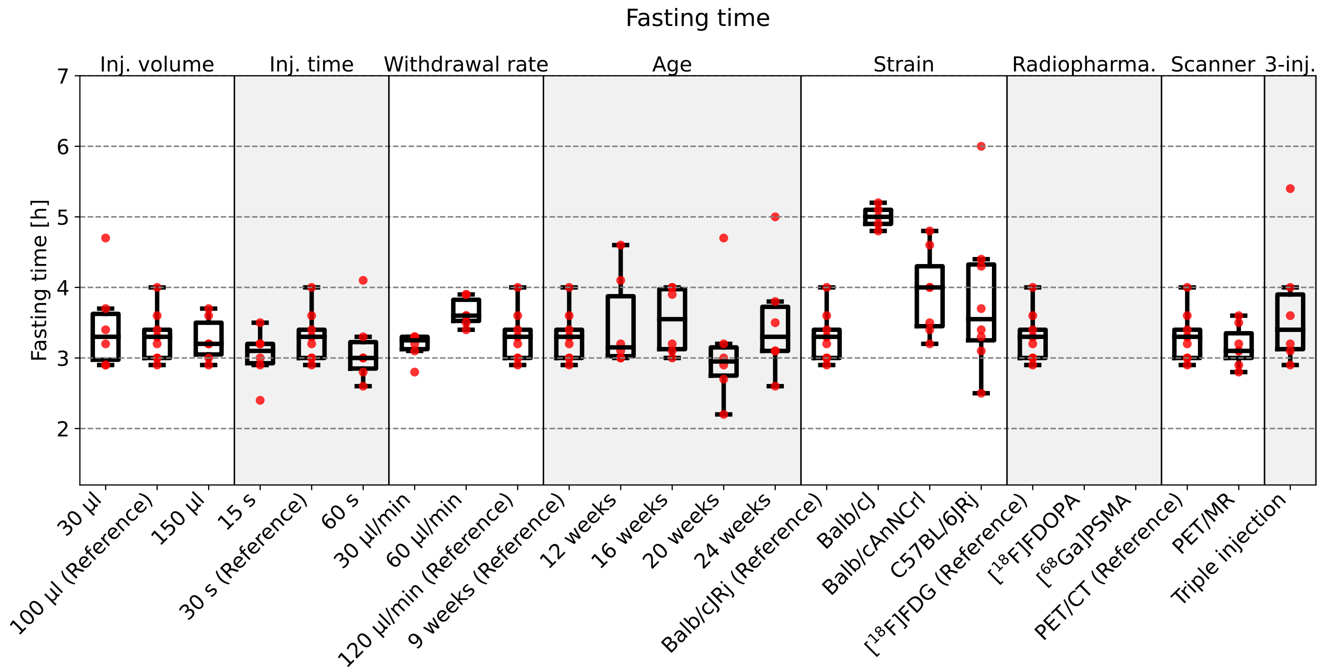

(a)

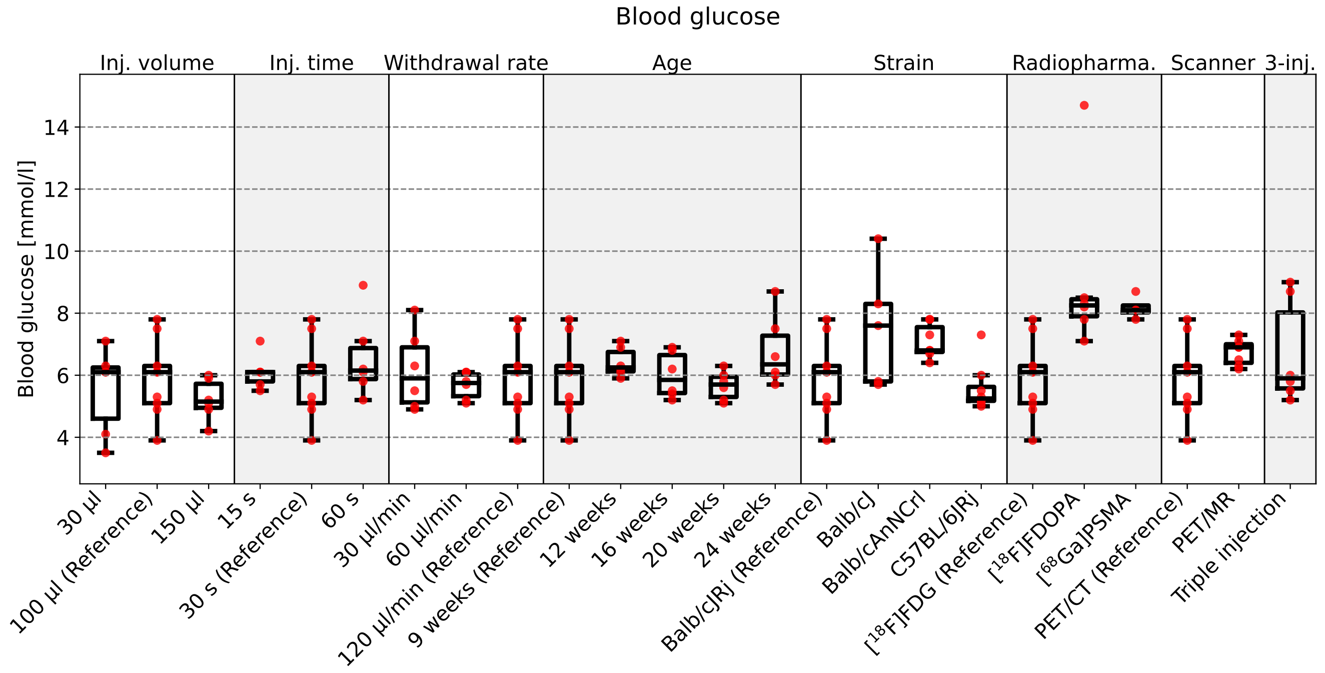

(b)

Figure S4: Distribution of fasting time (a) and blood glucose (b) of the mice for each experiment. Note that the reference distribution in each experiment correspond to the same data. In the boxplot, the horizontal line and the black box represent median and interquartile range (25th to 75th percentile), respectively, while the whiskers indicate the maximum and minimum data point up to 1.5 × interquartile range. Individual data points are shown with red dots.

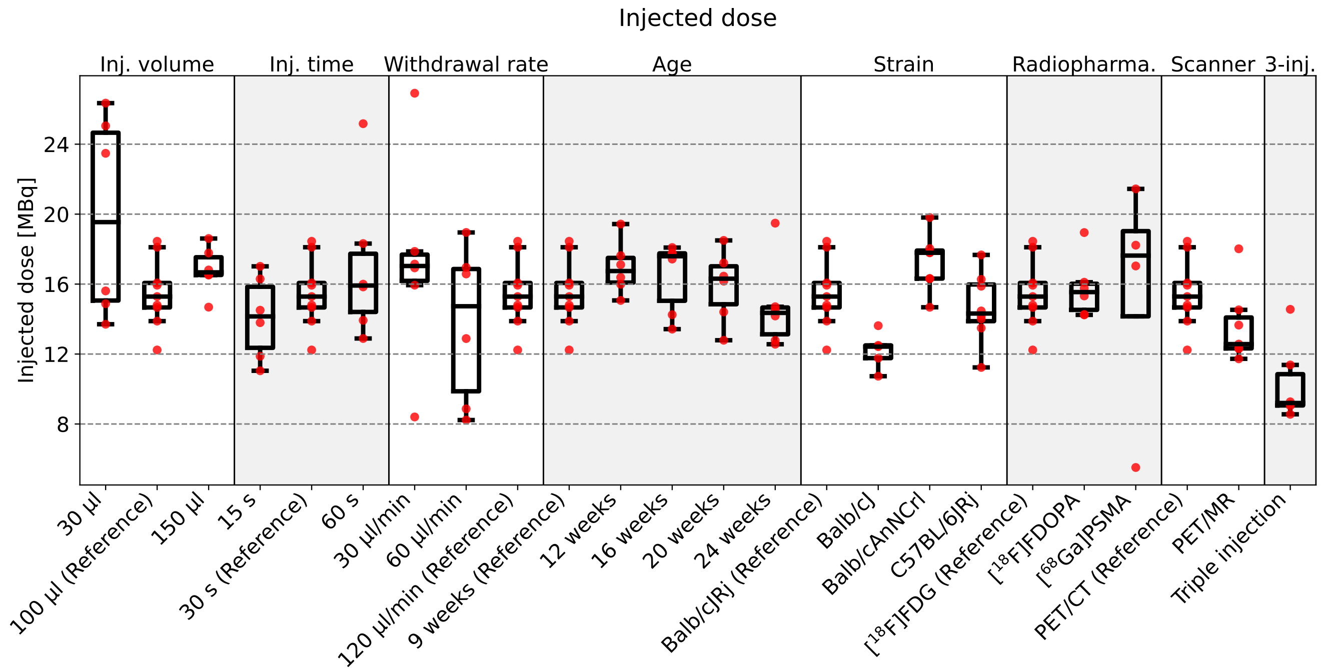

(a)

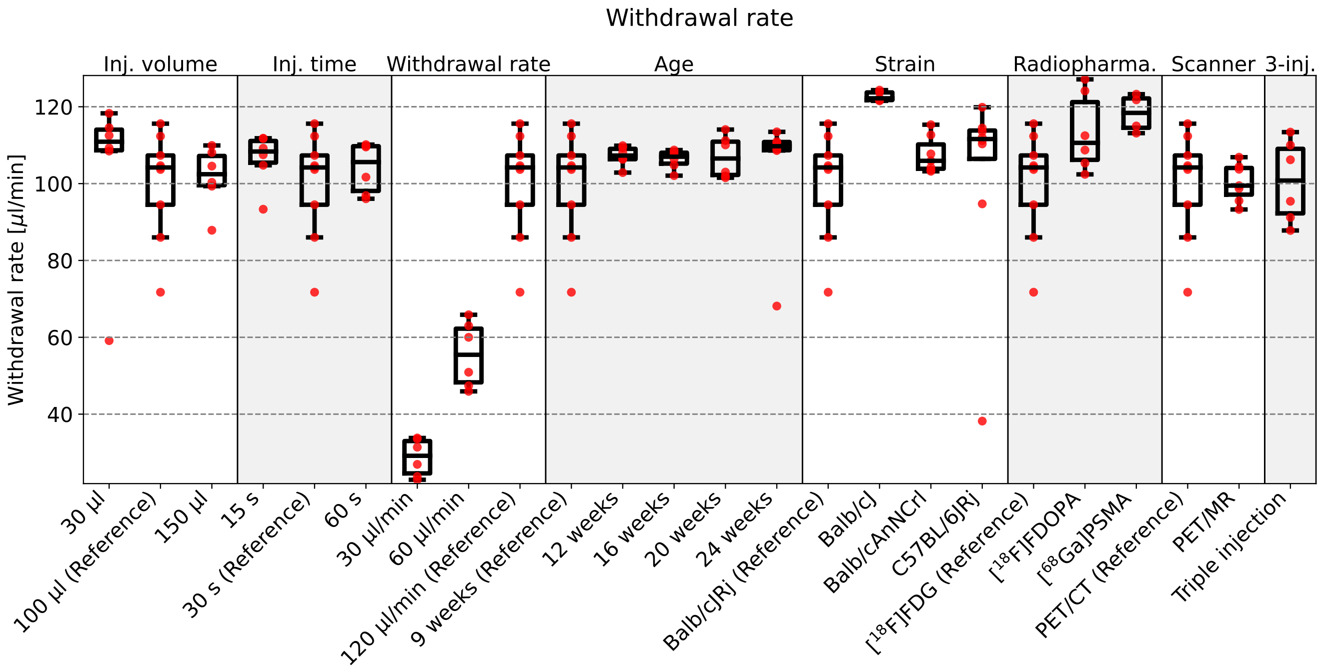

(b)

Figure S5: Distribution of injected dose (a) and arterial blood withdrawal rate (b) of the mice for each experiment. Note that the reference distribution in each experiment correspond to the same data. In the boxplot, the horizontal line and the black box represent median and interquartile range (25th to 75th percentile), respectively, while the whiskers indicate the maximum and minimum data point up to 1.5 $\times$ interquartile range. Individual data points are shown with red dots.

S8 Processing of the AIF, IDIF, and tissue curves

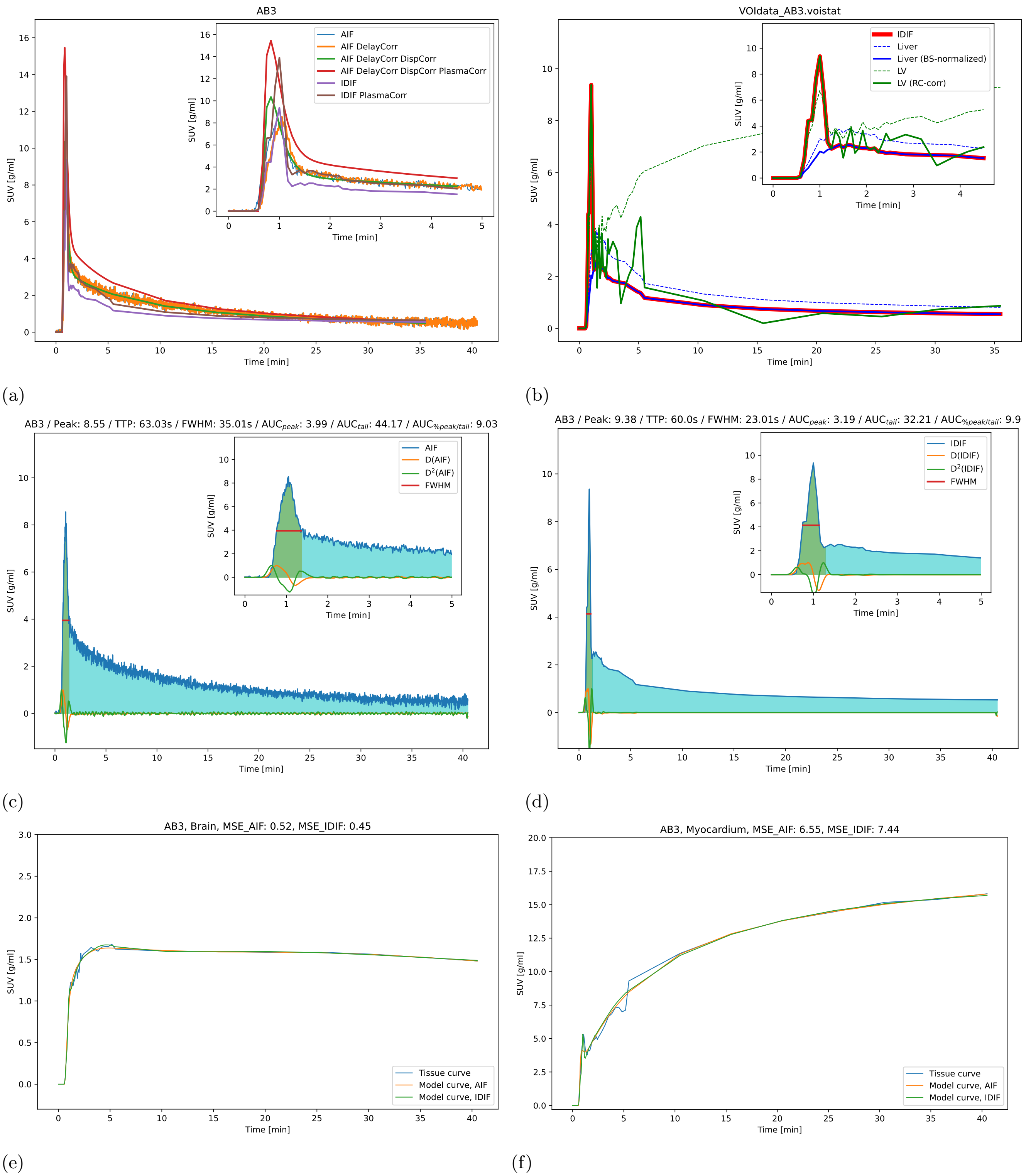

Figure S6: Processing and feature extraction of the AIF and IDIF curves and the tissue and model curves following from tracer kinetic modeling, exemplified for one representative mouse, AB3. (a) The processing steps for the AIF: Measured AIF, delay corrected AIF to the IDIF, dispersion corrected AIF, plasma corrected AIF and lastly the IDIF and plasma corrected IDIF. (b) The processing steps for the IDIF: Left ventricle (LV), LV corrected for recovery coefficient (RC), liver curve, liver normalized to the last blood sample (BS), and finally the IDIF curve comprising of the RC corrected (RC-corr) LV curve for early time frames, and the BS-normalized LV curve for late time frames. (c) - (d) Measured AIF and IDIF data, its first and second derivative, used for separating the peak from the tail region. The extracted features are indicated in the title for peak value, time to peak (TTP), full-width at half maximum (FWHM), area under curve (AUC) peak and tail and the percentage ratio between these. The FWHM is also visualized with a red horizontal line in the figures. (e) - (f) Measured tissue curves for brain and myocardium regions, with respective model curve originating from tracer kinetic modeling using AIF and IDIF, respectively.

S9 Injection volume

Table S1: AIF features and kinetic modeling results for different radiotracer injection volumes. P values are calculated for each group compared to the Reference group.

			30 μl (N = 6)			100 μl (Reference) (N = 9)		150 μl (N = 6)		
Feature	Tissue	Unit	Mean	CI	P	Mean	CI	Mean	CI	P
Peak		[g/ml]	8.592	0.699	n.s.	8.781	0.747	8.346	0.629	n.s.
TTP		[s]	63.359	4.253	n.s.	61.025	2.465	60.858	2.42	n.s.
FWHM		[s]	37.015	5.146	n.s.	35.237	3.078	38.516	1.515	n.s.
AUC peak		[g/ml · min]	4.536	0.741	n.s.	4.487	0.414	4.495	0.296	n.s.
AUC tail		[g/ml · min]	47.874	5.039	n.s.	43.468	3.298	42.614	4.701	n.s.
AUC ratio		[1/1]	0.094	0.007	n.s.	0.104	0.01	0.106	0.007	n.s.
K_1	Brain	[ml/min/ml]	0.198	0.032	n.s.	0.178	0.019	0.208	0.019	n.s.
k_2	Brain	[1/min]	0.511	0.055	0.0176	0.431	0.034	0.48	0.038	n.s.
k_3	Brain	[1/min]	0.091	0.015	n.s.	0.089	0.011	0.096	0.029	n.s.
k_4	Brain	[1/min]	0.022	0.003	n.s.	0.019	0.003	0.02	0.002	n.s.
vB	Brain	[1/1]	0.006	0.005	n.s.	0.002	0.001	0.001	0.001	n.s.
K_i	Brain	[ml/g/min]	0.029	0.005	n.s.	0.031	0.005	0.034	0.007	n.s.
$K_{i,Patlak}$	Brain	[ml/g/min]	0.018	0.002	n.s.	0.021	0.004	0.022	0.005	n.s.
K_1	Myocardium	[ml/min/ml]	0.678	0.571	n.s.	0.907	0.449	0.69	0.619	n.s.
k_2	Myocardium	[1/min]	1.731	2.959	n.s.	2.175	2.053	1.173	1.996	n.s.
k_3	Myocardium	[1/min]	0.412	0.626	n.s.	0.244	0.196	0.128	0.119	n.s.
k_4	Myocardium	[1/min]	0.013	0.012	n.s.	0.004	0.005	0.004	0.002	n.s.
vB	Myocardium	[1/1]	0.185	0.085	n.s.	0.121	0.052	0.19	0.073	n.s.
K_i	Myocardium	[ml/g/min]	0.211	0.091	n.s.	0.216	0.051	0.175	0.029	n.s.
$K_{i,Patlak}$	Myocardium	[ml/g/min]	0.132	0.042	n.s.	0.182	0.046	0.14	0.019	n.s.

TTP: time to peak, AUC: area under curve, CI: confidence interval, vB: fractional blood volume

Table S2: IDIF features and kinetic modeling results for different radiotracer injection volumes. P values are calculated for each group compared to the Reference group.

			30 μl (N = 6)			100 μl (Reference) (N = 9)		150 μl (N = 6)		
Feature	Tissue	Unit	Mean	CI	P	Mean	CI	Mean	CI	P
Peak		[g/ml]	8.84	1.158	n.s.	8.969	1.356	9.682	1.072	n.s.
TTP		[s]	59.167	2.749	n.s.	56.111	3.702	58.333	2.982	n.s.
FWHM		[s]	23.843	4.04	n.s.	28.123	3.451	28.178	4.37	n.s.
AUC peak		[g/ml · min]	3.101	0.622	n.s.	3.652	0.539	4.109	0.516	n.s.
AUC tail		[g/ml · min]	33.263	5.055	n.s.	29.777	4.236	30.502	3.993	n.s.
AUC ratio		[1/1]	0.093	0.008	0.0496	0.127	0.022	0.137	0.021	n.s.
K_1	Brain	[ml/min/ml]	0.285	0.06	n.s.	0.237	0.024	0.232	0.023	n.s.
k_2	Brain	[1/min]	0.526	0.078	0.0176	0.38	0.05	0.381	0.082	n.s.
k_3	Brain	[1/min]	0.137	0.031	n.s.	0.124	0.022	0.143	0.057	n.s.
k_4	Brain	[1/min]	0.049	0.006	n.s.	0.048	0.007	0.05	0.009	n.s.
vB	Brain	[1/1]	0.015	0.005	n.s.	0.014	0.005	0.014	0.005	n.s.
K_i	Brain	[ml/g/min]	0.059	0.016	n.s.	0.06	0.012	0.061	0.018	n.s.
$K_{i,Patlak}$	Brain	[ml/g/min]	0.02	0.006	n.s.	0.022	0.006	0.022	0.006	n.s.
K_1	Myocardium	[ml/min/ml]	1.405	0.765	n.s.	1.14	0.398	1.45	0.813	n.s.
k_2	Myocardium	[1/min]	4.268	3.582	n.s.	2.625	2.502	4.572	3.702	n.s.
k_3	Myocardium	[1/min]	1.419	1.272	n.s.	0.558	0.595	0.707	0.555	n.s.
k_4	Myocardium	[1/min]	1.69	2.973	n.s.	4.317	3.148	3.127	3.529	n.s.
vB	Myocardium	[1/1]	0.215	0.063	n.s.	0.221	0.047	0.201	0.033	n.s.
K_i	Myocardium	[ml/g/min]	0.366	0.246	n.s.	0.248	0.16	0.194	0.112	n.s.
$K_{i,Patlak}$	Myocardium	[ml/g/min]	0.179	0.094	n.s.	0.246	0.07	0.168	0.025	n.s.

TTP: time to peak, AUC: area under curve, CI: confidence interval, vB: fractional blood volume

S10 Injection time

Table S3: AIF features and kinetic modeling results for different radiotracer injection times. P values are calculated for each group compared to the Reference group.

			15 s (N = 6)			30 s (Reference) (N = 9)		60 s (N = 6)		
Feature	Tissue	Unit	Mean	CI	P	Mean	CI	Mean	CI	P
Peak		[g/ml]	10.455	0.597	0.0176	8.781	0.747	5.715	0.532	0.0004
TTP		[s]	50.854	2.902	0.0026	61.025	2.465	86.869	5.258	0.0004
FWHM		[s]	31.013	6.286	n.s.	35.237	3.078	65.193	3.906	0.0018
AUC peak		[g/ml · min]	5.026	1.177	n.s.	4.487	0.414	5.27	0.579	n.s.
AUC tail		[g/ml · min]	40.436	1.479	n.s.	43.468	3.298	43.768	3.162	n.s.
AUC ratio		[1/1]	0.125	0.03	n.s.	0.104	0.01	0.12	0.009	n.s.
K_1	Brain	[ml/min/ml]	0.205	0.029	n.s.	0.178	0.019	0.153	0.014	n.s.
k_2	Brain	[1/min]	0.475	0.052	n.s.	0.431	0.034	0.377	0.022	0.036
k_3	Brain	[1/min]	0.086	0.013	n.s.	0.089	0.011	0.085	0.019	n.s.
k_4	Brain	[1/min]	0.02	0.001	n.s.	0.019	0.003	0.016	0.002	n.s.
vB	Brain	[1/1]	0.007	0.004	n.s.	0.002	0.001	0	0	n.s.
K_i	Brain	[ml/g/min]	0.031	0.005	n.s.	0.031	0.005	0.028	0.005	n.s.
$K_{i,Patlak}$	Brain	[ml/g/min]	0.02	0.003	n.s.	0.021	0.004	0.02	0.004	n.s.
K_1	Myocardium	[ml/min/ml]	1.025	0.634	n.s.	0.907	0.449	0.672	0.429	n.s.
k_2	Myocardium	[1/min]	2.232	2.428	n.s.	2.175	2.053	1.553	1.675	n.s.
k_3	Myocardium	[1/min]	0.231	0.179	n.s.	0.244	0.196	0.266	0.167	n.s.
k_4	Myocardium	[1/min]	0.011	0.006	n.s.	0.004	0.005	0.006	0.009	n.s.
vB	Myocardium	[1/1]	0.153	0.093	n.s.	0.121	0.052	0.083	0.048	n.s.
K_i	Myocardium	[ml/g/min]	0.195	0.033	n.s.	0.216	0.051	0.176	0.027	n.s.
$K_{i,Patlak}$	Myocardium	[ml/g/min]	0.141	0.033	n.s.	0.182	0.046	0.153	0.017	n.s.

TTP: time to peak, AUC: area under curve, CI: confidence interval, vB: fractional blood volume

Table S4: IDIF features and kinetic modeling results for different radiotracer injection times. P values are calculated for each group compared to the Reference group.

			15 s (N = 6)			30 s (Reference) (N = 9)		60 s (N = 6)		
Feature	Tissue	Unit	Mean	CI	P	Mean	CI	Mean	CI	P
Peak		[g/ml]	12.376	2.243	n.s.	8.969	1.356	5.587	0.9	0.0048
TTP		[s]	46.667	1.886	0.0091	56.111	3.702	78.333	5.964	0.0016
FWHM		[s]	23.51	7.036	n.s.	28.123	3.451	41.684	4.473	0.0032
AUC peak		[g/ml · min]	4.359	0.925	n.s.	3.652	0.539	3.275	0.633	n.s.
AUC tail		[g/ml · min]	29.451	4.248	n.s.	29.777	4.236	33.563	3.773	n.s.
AUC ratio		[1/1]	0.15	0.029	n.s.	0.127	0.022	0.097	0.012	n.s.
K_1	Brain	[ml/min/ml]	0.232	0.035	n.s.	0.237	0.024	0.25	0.033	n.s.
k_2	Brain	[1/min]	0.412	0.063	n.s.	0.38	0.05	0.482	0.059	0.0496
k_3	Brain	[1/min]	0.121	0.017	n.s.	0.124	0.022	0.13	0.029	n.s.
k_4	Brain	[1/min]	0.044	0.004	n.s.	0.048	0.007	0.041	0.004	n.s.
vB	Brain	[1/1]	0.013	0.005	n.s.	0.014	0.005	0.017	0.012	n.s.
K_i	Brain	[ml/g/min]	0.055	0.016	n.s.	0.06	0.012	0.054	0.014	n.s.
$K_{i,Patlak}$	Brain	[ml/g/min]	0.02	0.005	n.s.	0.022	0.006	0.021	0.006	n.s.
K_1	Myocardium	[ml/min/ml]	1.159	0.475	n.s.	1.14	0.398	1.622	0.966	n.s.
k_2	Myocardium	[1/min]	3.961	3.558	n.s.	2.625	2.502	4.298	3.601	n.s.
k_3	Myocardium	[1/min]	1.025	0.964	n.s.	0.558	0.595	0.563	0.415	n.s.
k_4	Myocardium	[1/min]	1.674	2.943	n.s.	4.317	3.148	0.035	0.043	n.s.
vB	Myocardium	[1/1]	0.206	0.058	n.s.	0.221	0.047	0.157	0.096	n.s.
K_i	Myocardium	[ml/g/min]	0.351	0.125	n.s.	0.248	0.16	0.309	0.075	n.s.
$K_{i,Patlak}$	Myocardium	[ml/g/min]	0.174	0.063	n.s.	0.246	0.07	0.197	0.024	n.s.

TTP: time to peak, AUC: area under curve, CI: confidence interval, vB: fractional blood volume

S11 Withdrawal rate

Table S5: AIF features and kinetic modeling results for different arterial blood withdrawal rates. P values are calculated for each group compared to the Reference group.

			30 μl/min (N = 6)			60 μl/min (N = 6)			120 μl/min (Reference) (N = 9)	
Feature	Tissue	Unit	Mean	CI	P	Mean	CI	P	Mean	CI
Peak		[g/ml]	6.593	0.341	0.0004	7.881	0.86	n.s.	8.781	0.747
TTP		[s]	64.527	2.685	n.s.	63.193	2.631	n.s.	61.025	2.465
FWHM		[s]	44.352	6.108	0.0108	42.184	4.79	n.s.	35.237	3.078
AUC peak		[g/ml · min]	4.18	0.455	n.s.	4.572	0.712	n.s.	4.487	0.414
AUC tail		[g/ml · min]	44.628	4.771	n.s.	45.023	4.479	n.s.	43.468	3.298
AUC ratio		[1/1]	0.096	0.016	n.s.	0.101	0.011	n.s.	0.104	0.01
K_1	Brain	[ml/min/ml]	0.188	0.018	n.s.	0.17	0.016	n.s.	0.178	0.019
k_2	Brain	[1/min]	0.48	0.033	n.s.	0.451	0.031	n.s.	0.431	0.034
k_3	Brain	[1/min]	0.089	0.006	n.s.	0.084	0.011	n.s.	0.089	0.011
k_4	Brain	[1/min]	0.02	0.001	n.s.	0.02	0.004	n.s.	0.019	0.003
vB	Brain	[1/1]	0.006	0.005	n.s.	0.003	0.004	n.s.	0.002	0.001
K_i	Brain	[ml/g/min]	0.029	0.003	n.s.	0.027	0.004	n.s.	0.031	0.005
$K_{i,Patlak}$	Brain	[ml/g/min]	0.019	0.002	n.s.	0.018	0.003	n.s.	0.021	0.004
K_1	Myocardium	[ml/min/ml]	0.82	0.472	n.s.	0.497	0.216	n.s.	0.907	0.449
k_2	Myocardium	[1/min]	3.424	3.721	n.s.	0.613	0.881	n.s.	2.175	2.053
k_3	Myocardium	[1/min]	0.95	0.956	n.s.	0.162	0.071	n.s.	0.244	0.196
k_4	Myocardium	[1/min]	0.014	0.009	n.s.	0.018	0.007	0.012	0.004	0.005
vB	Myocardium	[1/1]	0.18	0.063	n.s.	0.201	0.081	n.s.	0.121	0.052
K_i	Myocardium	[ml/g/min]	0.239	0.069	n.s.	0.179	0.049	n.s.	0.216	0.051
$K_{i,Patlak}$	Myocardium	[ml/g/min]	0.175	0.067	n.s.	0.12	0.037	n.s.	0.182	0.046

TTP: time to peak, AUC: area under curve, CI: confidence interval, vB: fractional blood volume

Table S6: IDIF features and kinetic modeling results for different arterial blood withdrawal rates. P values are calculated for each group compared to the Reference group.

			30 μl/min (N = 6)			60 μl/min (N = 6)			120 μl/min (Reference) (N = 9)	
Feature	Tissue	Unit	Mean	CI	P	Mean	CI	P	Mean	CI
Peak		[g/ml]	8.659	1.376	n.s.	8.85	1.825	n.s.	8.969	1.356
TTP		[s]	59.167	1.491	n.s.	60	3.267	n.s.	56.111	3.702
FWHM		[s]	26.678	3.089	n.s.	38.349	14.369	n.s.	28.123	3.451
AUC peak		[g/ml · min]	3.187	0.328	n.s.	4.504	0.582	n.s.	3.652	0.539
AUC tail		[g/ml · min]	33.507	7.761	n.s.	34.908	4.497	n.s.	29.777	4.236
AUC ratio		[1/1]	0.102	0.024	n.s.	0.13	0.015	n.s.	0.127	0.022
K_1	Brain	[ml/min/ml]	0.242	0.031	n.s.	0.187	0.027	0.0496	0.237	0.024
k_2	Brain	[1/min]	0.436	0.057	n.s.	0.392	0.087	n.s.	0.38	0.05
k_3	Brain	[1/min]	0.129	0.025	n.s.	0.118	0.017	n.s.	0.124	0.022
k_4	Brain	[1/min]	0.049	0.003	n.s.	0.046	0.008	n.s.	0.048	0.007
vB	Brain	[1/1]	0.013	0.006	n.s.	0.017	0.003	n.s.	0.014	0.005
K_i	Brain	[ml/g/min]	0.055	0.013	n.s.	0.045	0.01	n.s.	0.06	0.012
$K_{i,Patlak}$	Brain	[ml/g/min]	0.018	0.003	n.s.	0.016	0.004	n.s.	0.022	0.006
K_1	Myocardium	[ml/min/ml]	0.6	0.129	0.0256	1.305	0.944	n.s.	1.14	0.398
k_2	Myocardium	[1/min]	0.057	0.03	n.s.	3.367	3.753	n.s.	2.625	2.502
k_3	Myocardium	[1/min]	0.287	0.277	n.s.	0.344	0.282	n.s.	0.558	0.595
k_4	Myocardium	[1/min]	2.852	3.212	n.s.	1.641	2.856	n.s.	4.317	3.148
vB	Myocardium	[1/1]	0.261	0.044	n.s.	0.219	0.082	n.s.	0.221	0.047
K_i	Myocardium	[ml/g/min]	0.389	0.153	n.s.	0.279	0.121	n.s.	0.248	0.16
$K_{i,Patlak}$	Myocardium	[ml/g/min]	0.23	0.111	n.s.	0.141	0.057	n.s.	0.246	0.07

TTP: time to peak, AUC: area under curve, CI: confidence interval, vB: fractional blood volume

S12 Mouse age

Table S7: AIF features and kinetic modeling results for different mouse ages. P values are calculated for each group compared to the Reference group.

			9 weeks (Reference) (N=9)		12 weeks (N=6)			16 weeks (N=6)			20 weeks (N=6)			24 weeks (N=6)		
Feature	Tissue	Unit	Mean	CI	Mean	CI	P	Mean	CI	P	Mean	CI	P	Mean	CI	P
Peak		[g/ml]	8.781	0.747	8.495	0.536	n.s.	8.735	0.92	n.s.	8.249	0.426	n.s.	8.401	0.696	n.s.
TTP		[s]	61.025	2.465	61.526	1.714	n.s.	61.859	0.971	n.s.	59.691	1.77	n.s.	58.858	1.815	n.s.
FWHM		[s]	35.237	3.078	40.183	3.08	n.s.	37.849	2.037	n.s.	38.849	2.711	n.s.	39.683	1.361	n.s.
AUC peak		[g/ml · min]	4.487	0.414	4.673	0.503	n.s.	4.601	0.683	n.s.	4.336	0.351	n.s.	4.61	0.417	n.s.
AUC tail		[g/ml · min]	43.468	3.298	45.534	4.169	n.s.	48.132	3.524	n.s.	45.813	5.71	n.s.	43.602	5.239	n.s.
AUC ratio		[1/1]	0.104	0.01	0.103	0.01	n.s.	0.095	0.007	n.s.	0.096	0.008	n.s.	0.107	0.008	n.s.
K_1	Brain	[ml/min/ml]	0.178	0.019	0.158	0.016	n.s.	0.156	0.01	n.s.	0.185	0.032	n.s.	0.18	0.039	n.s.
k_2	Brain	[1/min]	0.431	0.034	0.414	0.023	n.s.	0.426	0.013	n.s.	0.46	0.033	n.s.	0.473	0.037	n.s.
k_3	Brain	[1/min]	0.089	0.011	0.08	0.008	n.s.	0.083	0.009	n.s.	0.097	0.022	n.s.	0.109	0.016	n.s.
k_4	Brain	[1/min]	0.019	0.003	0.019	0.001	n.s.	0.018	0.003	n.s.	0.02	0.003	n.s.	0.018	0.004	n.s.
vB	Brain	[1/1]	0.002	0.001	0	0.001	n.s.	0	0.001	n.s.	0.001	0.001	n.s.	0	0	n.s.
K_i	Brain	[ml/g/min]	0.031	0.005	0.025	0.003	n.s.	0.025	0.003	n.s.	0.033	0.01	n.s.	0.034	0.01	n.s.
$K_{i,Patlak}$	Brain	[ml/g/min]	0.021	0.004	0.017	0.002	n.s.	0.017	0.003	n.s.	0.022	0.008	n.s.	0.023	0.007	n.s.
K_1	Myocardium	[ml/min/ml]	0.907	0.449	0.734	0.324	n.s.	0.837	0.588	n.s.	0.792	0.414	n.s.	0.733	0.401	n.s.
k_2	Myocardium	[1/min]	2.175	2.053	2.883	2.922	n.s.	2.942	3.215	n.s.	1.621	1.739	n.s.	1.654	1.833	n.s.
k_3	Myocardium	[1/min]	0.244	0.196	0.975	0.895	n.s.	0.458	0.45	n.s.	0.259	0.267	n.s.	0.31	0.297	n.s.
k_4	Myocardium	[1/min]	0.004	0.005	1.15	2.045	n.s.	0.016	0.005	0.012	0.007	0.006	n.s.	0.008	0.004	n.s.
vB	Myocardium	[1/1]	0.121	0.052	0.099	0.059	n.s.	0.134	0.076	n.s.	0.128	0.063	n.s.	0.1	0.057	n.s.
K_i	Myocardium	[ml/g/min]	0.216	0.051	0.195	0.063	n.s.	0.181	0.032	n.s.	0.223	0.107	n.s.	0.233	0.066	n.s.
$K_{i,Patlak}$	Myocardium	[ml/g/min]	0.182	0.046	0.134	0.034	n.s.	0.128	0.034	n.s.	0.182	0.096	n.s.	0.196	0.054	n.s.

TTP: time to peak, AUC: area under curve, CI: confidence interval, vB: fractional blood volume

Table S8: IDIF features and kinetic modeling results for different mouse ages. P values are calculated for each group compared to the Reference group.

			9 weeks (Reference) (N=9)		12 weeks (N=6)			16 weeks (N=6)			20 weeks (N=6)			24 weeks (N=6)		
Feature	Tissue	Unit	Mean	CI	Mean	CI	P	Mean	CI	P	Mean	CI	P	Mean	CI	P
Peak		[g/ml]	8.969	1.356	7.801	0.868	n.s.	8.413	1.029	n.s.	7.438	0.813	n.s.	7.616	0.41	n.s.
TTP		[s]	56.111	3.702	60	2.31	n.s.	60.833	1.49	n.s.	60	2.311	n.s.	60.833	2.749	n.s.
FWHM		[s]	28.123	3.451	32.514	4.427	n.s.	34.014	3.135	n.s.	37.349	8.679	n.s.	31.513	5.503	n.s.
AUC peak		[g/ml · min]	3.652	0.539	3.771	0.495	n.s.	4.555	0.474	n.s.	4.033	0.853	n.s.	3.879	0.659	n.s.
AUC tail		[g/ml · min]	29.777	4.236	28.908	3.24	n.s.	32.656	3.976	n.s.	31.017	6.777	n.s.	24.074	4.632	n.s.
AUC ratio		[1/1]	0.127	0.022	0.131	0.01	n.s.	0.142	0.02	n.s.	0.137	0.033	n.s.	0.171	0.041	n.s.
K_1	Brain	[ml/min/ml]	0.237	0.024	0.195	0.027	n.s.	0.178	0.009	0.0496	0.241	0.036	n.s.	0.231	0.039	n.s.
k_2	Brain	[1/min]	0.38	0.05	0.3	0.071	0.036	0.308	0.041	n.s.	0.414	0.079	n.s.	0.314	0.034	n.s.
k_3	Brain	[1/min]	0.124	0.022	0.104	0.02	n.s.	0.103	0.018	n.s.	0.142	0.024	n.s.	0.152	0.026	n.s.
k_4	Brain	[1/min]	0.048	0.007	0.05	0.007	n.s.	0.043	0.006	n.s.	0.047	0.005	n.s.	0.047	0.01	n.s.
vB	Brain	[1/1]	0.014	0.005	0.019	0.012	n.s.	0.016	0.007	n.s.	0.015	0.005	n.s.	0.015	0.007	n.s.
K_i	Brain	[ml/g/min]	0.06	0.012	0.051	0.011	n.s.	0.045	0.007	n.s.	0.066	0.027	n.s.	0.078	0.026	n.s.
$K_{i,Patlak}$	Brain	[ml/g/min]	0.022	0.006	0.017	0.002	n.s.	0.018	0.004	n.s.	0.025	0.011	n.s.	0.032	0.01	n.s.
K_1	Myocardium	[ml/min/ml]	1.14	0.398	0.646	0.287	0.0496	0.633	0.366	n.s.	0.953	0.55	n.s.	0.687	0.095	n.s.
k_2	Myocardium	[1/min]	2.625	2.502	0.409	0.675	n.s.	1.69	2.974	n.s.	1.695	2.972	n.s.	0.544	0.945	0.0496
k_3	Myocardium	[1/min]	0.558	0.595	0.123	0.135	n.s.	0.507	0.835	n.s.	0.72	0.766	n.s.	1.482	1.893	n.s.
k_4	Myocardium	[1/min]	4.317	3.148	6.328	3.581	n.s.	3.279	3.674	n.s.	3.976	3.207	n.s.	7.579	2.756	n.s.
vB	Myocardium	[1/1]	0.221	0.047	0.193	0.074	n.s.	0.258	0.053	n.s.	0.225	0.072	n.s.	0.188	0.041	n.s.
K_i	Myocardium	[ml/g/min]	0.248	0.16	0.132	0.105	n.s.	0.214	0.117	n.s.	0.303	0.223	n.s.	0.256	0.234	n.s.
$K_{i,Patlak}$	Myocardium	[ml/g/min]	0.246	0.07	0.187	0.06	n.s.	0.166	0.043	n.s.	0.251	0.152	n.s.	0.316	0.096	n.s.

TTP: time to peak, AUC: area under curve, CI: confidence interval, vB: fractional blood volume

S13 Mouse strain

Table S9: AIF features and kinetic modeling results for different mouse strains. P values are calculated for each group compared to the Reference group.

			Balb/cJRj (Reference) (N=9)		Balb/cJ (N=5)			Balb/cAnNCrl (N=5)			C57BL/6JRj (N=8)		
Feature	Tissue	Unit	Mean	CI	Mean	CI	P	Mean	CI	P	Mean	CI	P
Peak		[g/ml]	8.781	0.747	9.969	0.801	n.s.	9.337	1.716	n.s.	6.98	0.869	0.0152
TTP		[s]	61.025	2.465	59.825	1.023	n.s.	61.168	1.452	n.s.	61.9	2.177	n.s.
FWHM		[s]	35.237	3.078	37.616	2.392	n.s.	38.302	1.356	n.s.	36.765	1.724	n.s.
AUC peak		[g/ml · min]	4.487	0.414	5.521	0.324	0.007	5.232	1.272	n.s.	3.662	0.466	0.0206
AUC tail		[g/ml · min]	43.468	3.298	59.933	3.245	0.001	49.983	3.937	n.s.	49.492	5.979	n.s.
AUC ratio		[1/1]	0.104	0.01	0.092	0.005	n.s.	0.103	0.021	n.s.	0.075	0.006	0.0003
K_1	Brain	[ml/min/ml]	0.178	0.019	0.165	0.027	n.s.	0.148	0.019	n.s.	0.17	0.021	n.s.
k_2	Brain	[1/min]	0.431	0.034	0.404	0.032	n.s.	0.385	0.034	n.s.	0.418	0.022	n.s.
k_3	Brain	[1/min]	0.089	0.011	0.053	0.012	0.007	0.084	0.005	n.s.	0.073	0.007	n.s.
k_4	Brain	[1/min]	0.019	0.003	0.015	0.003	n.s.	0.018	0.003	n.s.	0.017	0.005	n.s.
vB	Brain	[1/1]	0.002	0.001	0.005	0.002	0.042	0	0	n.s.	0.001	0.001	n.s.
K_i	Brain	[ml/g/min]	0.031	0.005	0.019	0.004	0.019	0.026	0.002	n.s.	0.025	0.005	n.s.
$K_{i,Patlak}$	Brain	[ml/g/min]	0.021	0.004	0.013	0.003	0.042	0.018	0.002	n.s.	0.018	0.006	n.s.
K_1	Myocardium	[ml/min/ml]	0.907	0.449	1.037	0.765	n.s.	1.32	0.198	n.s.	0.715	0.39	n.s.
k_2	Myocardium	[1/min]	2.175	2.053	2.209	2.328	n.s.	4.494	0.974	n.s.	1.708	2.289	n.s.
k_3	Myocardium	[1/min]	0.244	0.196	0.234	0.173	n.s.	0.333	0.122	n.s.	0.321	0.341	n.s.
k_4	Myocardium	[1/min]	0.004	0.005	0.002	0.002	n.s.	0.006	0.004	n.s.	0.009	0.008	n.s.
vB	Myocardium	[1/1]	0.121	0.052	0.138	0.099	n.s.	0.003	0.004	0.0021	0.139	0.058	n.s.
K_i	Myocardium	[ml/g/min]	0.216	0.051	0.191	0.026	n.s.	0.089	0.027	0.0033	0.206	0.06	n.s.
$K_{i,Patlak}$	Myocardium	[ml/g/min]	0.182	0.046	0.157	0.022	n.s.	0.079	0.029	0.0079	0.165	0.046	n.s.

TTP: time to peak, AUC: area under curve, CI: confidence interval, vB: fractional blood volume

Table S10: IDIF features and kinetic modeling results for different mouse strains. P values are calculated for each group compared to the Reference group.

			Balb/cJRj (Reference) (N=9)		Balb/cJ (N=5)			Balb/cAnNCrl (N=5)			C57BL/6JRj (N=8)		
Feature	Tissue	Unit	Mean	CI	Mean	CI	P	Mean	CI	P	Mean	CI	P
Peak		[g/ml]	8.969	1.356	11.494	1.423	n.s.	7.763	1.253	n.s.	6.88	0.834	0.0464
TTP		[s]	56.111	3.702	58.199	2.314	n.s.	59.285	2.367	n.s.	60.625	2.705	n.s.
FWHM		[s]	28.123	3.451	27.812	2.85	n.s.	35.015	3.779	0.0341	34.764	6.361	n.s.
AUC peak		[g/ml · min]	3.652	0.539	4.92	0.591	0.042	4.067	0.99	n.s.	3.719	0.656	n.s.
AUC tail		[g/ml · min]	29.777	4.236	41.76	4.983	0.007	29.519	4.091	n.s.	30.769	6.018	n.s.
AUC ratio		[1/1]	0.127	0.022	0.119	0.015	n.s.	0.137	0.026	n.s.	0.131	0.031	n.s.
K_1	Brain	[ml/min/ml]	0.237	0.024	0.182	0.017	n.s.	0.202	0.031	n.s.	0.217	0.025	n.s.
k_2	Brain	[1/min]	0.38	0.05	0.302	0.021	n.s.	0.295	0.035	0.0115	0.293	0.072	n.s.
k_3	Brain	[1/min]	0.124	0.022	0.061	0.02	0.007	0.117	0.011	n.s.	0.084	0.016	0.0274
k_4	Brain	[1/min]	0.048	0.007	0.029	0.004	0.001	0.046	0.004	n.s.	0.051	0.009	n.s.
vB	Brain	[1/1]	0.014	0.005	0.016	0.003	n.s.	0.014	0.005	n.s.	0.02	0.004	n.s.
K_i	Brain	[ml/g/min]	0.06	0.012	0.03	0.009	0.012	0.058	0.009	n.s.	0.054	0.017	n.s.
$K_{i,Patlak}$	Brain	[ml/g/min]	0.022	0.006	0.015	0.004	n.s.	0.023	0.004	n.s.	0.023	0.014	n.s.
K_1	Myocardium	[ml/min/ml]	1.14	0.398	0.976	0.389	n.s.	1.327	0.543	n.s.	0.869	0.368	n.s.
k_2	Myocardium	[1/min]	2.625	2.502	3.372	3.196	n.s.	4.739	2.855	n.s.	1.276	2.285	n.s.
k_3	Myocardium	[1/min]	0.558	0.595	1.425	1.525	n.s.	1.144	0.708	n.s.	1.115	1.23	n.s.
k_4	Myocardium	[1/min]	4.317	3.148	0.017	0.01	n.s.	1.402	2.513	n.s.	7.171	2.881	n.s.
vB	Myocardium	[1/1]	0.221	0.047	0.224	0.05	n.s.	0.179	0.075	n.s.	0.245	0.048	n.s.
K_i	Myocardium	[ml/g/min]	0.248	0.16	0.338	0.087	n.s.	0.225	0.09	n.s.	0.383	0.21	n.s.
$K_{i,Patlak}$	Myocardium	[ml/g/min]	0.246	0.07	0.211	0.036	n.s.	0.128	0.058	n.s.	0.252	0.115	n.s.

TTP: time to peak, AUC: area under curve, CI: confidence interval, vB: fractional blood volume

S14 Radiopharmaceutical

Table S11: AIF features and kinetic modeling results for different radiopharmaceuticals. P values are calculated for each group compared to the Reference group.

			[18F]FDG (Reference) (N = 9)		[18F]FDOPA (N = 6)			[68Ga]PSMA-617 (N = 4)		
Feature	Tissue	Unit	Mean	CI	Mean	CI	P	Mean	CI	P
Peak		[g/ml]	8.781	0.747	8.61	0.797	n.s.	8.902	1.494	n.s.
TTP		[s]	61.025	2.465	61.526	1.007	n.s.	62.026	4.999	n.s.
FWHM		[s]	35.237	3.078	39.683	1.829	0.0446	36.015	4.999	n.s.
AUC peak		[g/ml · min]	4.487	0.414	4.626	0.337	n.s.	4.986	0.217	n.s.
AUC tail		[g/ml · min]	43.468	3.298	26.61	2.562	0.0004	38.608	5.708	n.s.
AUC ratio		[1/1]	0.104	0.01	0.176	0.017	0.0008	0.132	0.017	n.s.
K_1	Brain	[ml/min/ml]	0.178	0.019	0.086	0.011	0.0004	0.048	0.017	0.0028
k_2	Brain	[1/min]	0.431	0.034	1.176	0.264	0.0004	1.196	0.376	0.0028
k_3	Brain	[1/min]	0.089	0.011	0.166	0.015	0.0004	0.072	0.013	n.s.
k_4	Brain	[1/min]	0.019	0.003	0.038	0.004	0.0004	0.083	0.01	0.0028
vB	Brain	[1/1]	0.002	0.001	0.004	0.002	n.s.	0.005	0.004	0.0336
K_i	Brain	[ml/g/min]	0.031	0.005	0.011	0.001	0.0004	0.003	0.001	0.0028
$K_{i,Patlak}$	Brain	[ml/g/min]	0.021	0.004	0.004	0	0.0004	0	0	0.0028
K_1	Myocardium	[ml/min/ml]	0.907	0.449	1.595	0.248	n.s.	1.036	0.245	n.s.
k_2	Myocardium	[1/min]	2.175	2.053	4.379	0.543	n.s.	3.75	0.829	n.s.
k_3	Myocardium	[1/min]	0.244	0.196	0.039	0.015	0.0176	0.012	0.004	0.0028
k_4	Myocardium	[1/min]	0.004	0.005	0.06	0.006	0.0004	0.039	0.005	0.0028
vB	Myocardium	[1/1]	0.121	0.052	0.034	0.014	n.s.	0.043	0.019	n.s.
K_i	Myocardium	[ml/g/min]	0.216	0.051	0.013	0.003	0.0004	0.003	0	0.0028
$K_{i,Patlak}$	Myocardium	[ml/g/min]	0.182	0.046	0.002	0	0.0004	0.001	0	0.0028

TTP: time to peak, AUC: area under curve, CI: confidence interval, vB: fractional blood volume

S15 PET scanner

Table S12: AIF features and kinetic modeling results for different PET scanners. P values are calculated for the PET/MR group compared to the PET/CT (Reference) group.

			PET/CT (Reference) N=9		PET/MR N=7		
Feature	Tissue	Unit	Mean	CI	Mean	CI	P
Peak		[g/ml]	8.781	0.747	6.024	0.62	0.0002
TTP		[s]	61.025	2.465	63.455	1.179	n.s.
FWHM		[s]	35.237	3.078	50.878	19.833	n.s.
AUC peak		[g/ml · min]	4.487	0.414	4.247	0.906	n.s.
AUC tail		[g/ml · min]	43.468	3.298	45.821	4.983	n.s.
AUC ratio		[1/1]	0.104	0.01	0.097	0.03	n.s.
K_1	Brain	[ml/min/ml]	0.178	0.019	0.165	0.039	n.s.
k_2	Brain	[1/min]	0.431	0.034	0.411	0.088	n.s.
k_3	Brain	[1/min]	0.089	0.011	0.08	0.022	n.s.
k_4	Brain	[1/min]	0.019	0.003	0.014	0.004	n.s.
vB	Brain	[1/1]	0.002	0.001	0.004	0.005	n.s.
K_i	Brain	[ml/g/min]	0.031	0.005	0.027	0.008	n.s.
$K_{i,Patlak}$	Brain	[ml/g/min]	0.021	0.004	0.019	0.003	n.s.
K_1	Myocardium	[ml/min/ml]	0.907	0.449	1.238	0.596	n.s.
k_2	Myocardium	[1/min]	2.175	2.053	5.755	3.632	n.s.
k_3	Myocardium	[1/min]	0.244	0.196	0.862	0.569	n.s.
k_4	Myocardium	[1/min]	0.004	0.005	0.007	0.005	n.s.
vB	Myocardium	[1/1]	0.121	0.052	0.102	0.082	n.s.
K_i	Myocardium	[ml/g/min]	0.216	0.051	0.208	0.05	n.s.
$K_{i,Patlak}$	Myocardium	[ml/g/min]	0.182	0.046	0.164	0.036	n.s.

TTP: time to peak, AUC: area under curve, CI: confidence interval, vB: fractional blood volume

Table S13: IDIF features and kinetic modeling results for different PET scanners. P values are calculated for the PET/MR group compared to the PET/CT (Reference) group.

			PET/CT (Reference) N=9		PET/MR N=7		
Feature	Tissue	Unit	Mean	CI	Mean	CI	P
Peak		[g/ml]	8.969	1.356	6.285	0.864	0.0052
TTP		[s]	56.111	3.702	59.286	2.366	n.s.
FWHM		[s]	28.123	3.451	39.302	10.745	n.s.
AUC peak		[g/ml · min]	3.652	0.539	3.457	0.667	n.s.
AUC tail		[g/ml · min]	29.777	4.236	23.473	2.299	n.s.
AUC ratio		[1/1]	0.127	0.022	0.152	0.038	n.s.
K_1	Brain	[ml/min/ml]	0.237	0.024	0.231	0.036	n.s.
k_2	Brain	[1/min]	0.38	0.05	0.247	0.053	0.0052
k_3	Brain	[1/min]	0.124	0.022	0.091	0.032	0.0311
k_4	Brain	[1/min]	0.048	0.007	0.047	0.01	n.s.
vB	Brain	[1/1]	0.014	0.005	0.007	0.005	n.s.
K_i	Brain	[ml/g/min]	0.06	0.012	0.062	0.019	n.s.
$K_{i,Patlak}$	Brain	[ml/g/min]	0.022	0.006	0.025	0.004	n.s.
K_1	Myocardium	[ml/min/ml]	1.14	0.398	0.637	0.106	n.s.
k_2	Myocardium	[1/min]	2.625	2.502	0.039	0.028	n.s.
k_3	Myocardium	[1/min]	0.558	0.595	0.032	0.038	n.s.
k_4	Myocardium	[1/min]	4.317	3.148	6.985	3.266	n.s.
vB	Myocardium	[1/1]	0.221	0.047	0.183	0.062	n.s.
K_i	Myocardium	[ml/g/min]	0.248	0.16	0.122	0.104	n.s.
$K_{i,Patlak}$	Myocardium	[ml/g/min]	0.246	0.07	0.277	0.069	n.s.

TTP: time to peak, AUC: area under curve, CI: confidence interval, vB: fractional blood volume

S16 Triple injection repeatability

Table S14: AIF features for the triple injection repeatability. The statistical comparison for injection 2 and 3 are done relative the first (Reference) injection.

		Injection 1 (Reference)		Injection 2			Injection 3		
Feature	Unit	Mean	CI	Mean	CI	P	Mean	CI	P
Peak	[g/ml]	7.258	0.441	7.823	0.774	n.s.	7.959	1.239	n.s.
TTP	[s]	64.5	3.09	65.333	0.754	n.s.	65.5	1.584	n.s.
FWHM	[s]	35.667	3.156	32.333	4.045	n.s.	33.833	2.749	n.s.
AUC peak	[g/ml · min]	3.578	0.226	3.569	0.229	n.s.	3.678	0.415	n.s.
AUC tail	[g/ml · min]	23.134	1.229	24.19	3.341	n.s.	26.66	5.782	n.s.
AUC ratio	[1/1]	0.156	0.014	0.151	0.018	n.s.	0.142	0.013	n.s.

TTP: time to peak, AUC: area under curve, CI: confidence interval

S17 Peristaltic pump withdrawal rate

The withdrawal rate of the peristaltic pump was calibrated daily by measuring the weight of a one-minute withdrawal of saline, and adjusting the withdrawal speed to the actual withdrawn volume. The saline was withdrawn from a falcon tube using a short, 10 cm catheter. In order to evaluate the actual withdrawal rate of blood under more realistic experimental conditions, the weight of withdrawn blood was carefully measured at the end of each PET experiment for each mouse, as described in Section S3. For three of the mice from the PET/MR experiments, where long tubing was utilized, three additional withdrawal measurements were performed to evaluate the impact of the tubing length on the actual withdrawal rate. Briefly, once the three late manual blood samples following each PET experiment had been taken, and while the mouse was still in deep anesthesia, catheter D (Figure S1) was disconnected from the peristaltic pump and blood was allowed to drip into a pre-weighed eppendorf tube for 60 seconds without the peristaltic pump aiding with blood withdrawal. This process was repeated three times. Subsequently, the 80 cm catheter B was cut to 30 cm, corresponding to the PET/CT setup, and reconnected to the radiation detector and to the peristaltic pump. Again, the blood was allowed to drip into a pre-weighed eppendorf tube for 30 s, first with the peristaltic pump switched on (3 measurements), and lastly, with catheter D (Figure S1) disconnected from the peristaltic pump (3 measurements). In this way, the withdrawal rate was measured under 4 experimental setups for each mouse. One of the three mice died after the fourth blood sample. The measured withdrawal rate was calculated as the ratio of the weight of the net content of each eppendorf tube to the withdrawal time.

The results from the withdrawal rate experiments are shown in Figure S7. For long catheters, the withdrawal rate was measured to 105.0 $\pm$ 1.3 μl/min (n=9) and 24.5 $\pm$ 2.1 μl/min (n=7) with and without the peristaltic pump, respectively. The corresponding measurements for short catheters were 116.9 $\pm$ 4.9 μl/min (n=6) and 59.1 $\pm$ 8.0 μl/min (n=6). All differences were significant ($P < 0.05$).

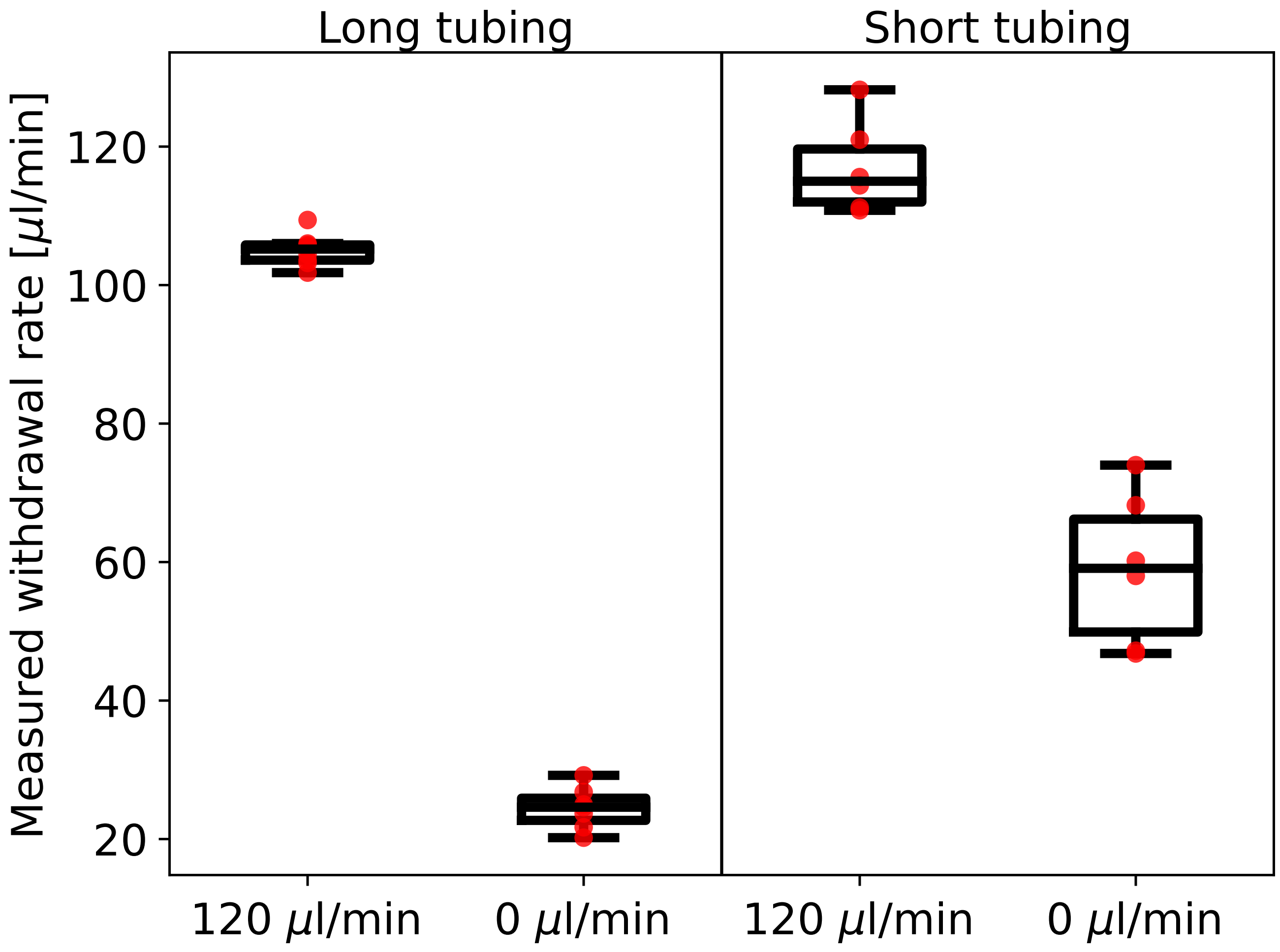

Figure S7: Measured arterial blood withdrawal rate for three mice using long and short tubings, corresponding to the PET/MR and PET/CT setup, respectively, with (120 μl/min) and without (0 μl/min) withdrawal pump. In the boxplot, the horizontal line and the black box represent median and interquartile range (25th to 75th percentile), respectively, while the whiskers indicate the maximum and minimum data point up to 1.5 $\times$ interquartile range. Individual data points are shown with red dots.

S18 Dispersion correction of the AIF

As pointed out in the Discussion section of the main paper, the AIF peak of the PET/MR scans were significantly lower due to increased dispersion in the longer tubing, compared to PET/CT. We investigated the impact of dispersion correction on the PET/MR AIF data. Briefly, the measured blood signal, $g(t)$, will be affected by dispersion, which can be modeled as a convolution of the true AIF, $C_A(t)$, and a dispersion function, $d(t)$ as [6]:

$$g(t) = C_A(t) \otimes d(t). \tag{1}$$

A mono-exponential dispersion model may be assumed [6]:

$$d(t) = \frac{1}{\tau} e^{\frac{-t}{\tau}}, \tag{2}$$

where τ is the dispersion constant. To create a dispersion corrected AIF, Equation 1 was implemented in the optimization step of the parametric model fit to the measured AIF data [5], as described in the main paper, as well as in [7].

Figure S6 shows the group averaged parametric fits to the AIF curves for varying PET scanners, where the PET/MR data was corrected for dispersion, as described above. Compared to Figure 9a in the main paper, the peak of the PET/MR data now aligns well with the PET/CT data. This indicates that dispersion correction of the measured AIF data could potentially compensate for the dispersion effect that is affecting the PET/MR data due to the significantly longer tubing, compared to the PET/CT setup.

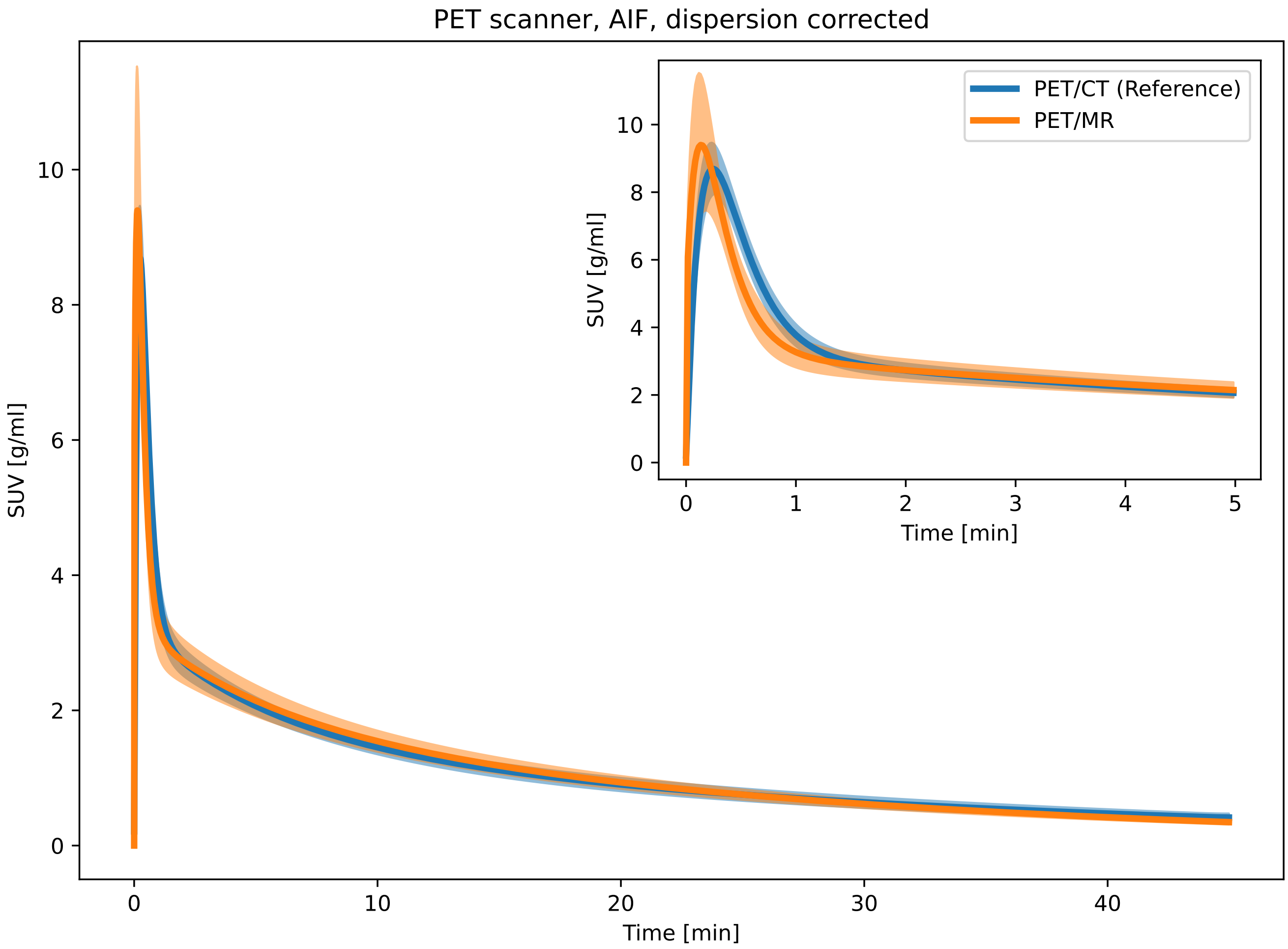

Figure S8: Parametric fit of the AIF curves for varying PET scanners. The PET/MR data was corrected for dispersion. The line and the shaded area represent the mean curve and the 95% confidence interval, respectively.

S19 Tracer kinetic modeling validation

Tracer kinetic modeling was implemented in pyPET, an in-house developed Python library for tracer kinetic modeling, available at https://github.com/Kuttner/pyPET. The pyPET library was validated against a commercially available kinetic modeling software package (PMOD v. 4.002, Bruker Corporation, MA, USA). Briefly, mice from the 8-week, 16-week, and 24-week age groups were included in the validation, totaling 21 mouse samples. Tracer kinetic modeling was performed using tissue curves from the brain and myocardium regions, along with the fully corrected arterial plasma input function, in both software packages. The resulting kinetic parameters were compared using scatter plots and linear regression. Both software packages implemented a non-linear least-squares algorithm to fit the model parameters. It is well known that such optimizers are sensitive to factors such as initial conditions, particularly when dealing with noisy data [8]. To address this, in addition to comparing all data, samples with kinetic modeling parameters exceeding three scaled median absolute deviations from the median were identified as outliers and removed to improve the comparison.

Figures S9 and S10 present the results with all samples and with outliers removed, respectively. The majority of data points align closely with the 45-degree line for all kinetic modeling parameters, indicating strong agreement between the pyPET and PMOD (Figure S9). Both software packages produced a few outlier samples where the kinetic modeling parameters deviated significantly from the main distribution, shown as off-diagonal data points. pyPET tended to produce fewer outlier samples for K_1, k_2, k_3, and k_4 (Figure S9(a)-(e)) and generated more vB data points greater than zero compared to PMOD (Figure S9(f)). The outliers influenced the linear regression fit lines in Figure S9. After removing outliers, the linear regression fit lines achieved a slope close to unity for all parameters (Figure S10(a)-(e)). An exception was observed for vB where many values were close to zero. Applying the outlier removal criterion in this case introduced a bias in the data distribution, resulting in a less reliable linear fit after outlier removal (Figure S10(f)).

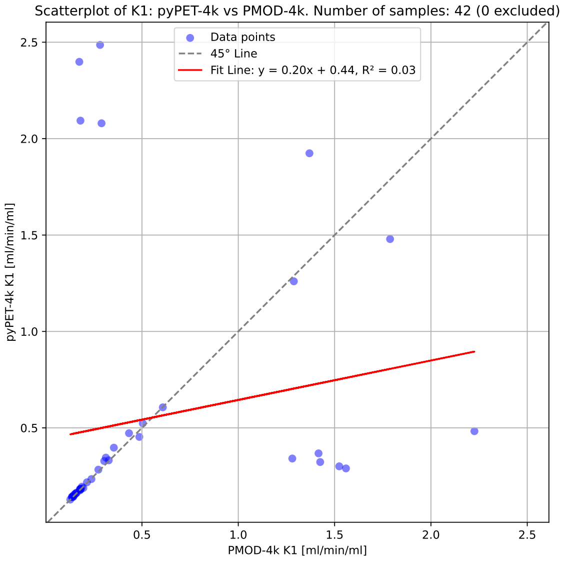

(a)

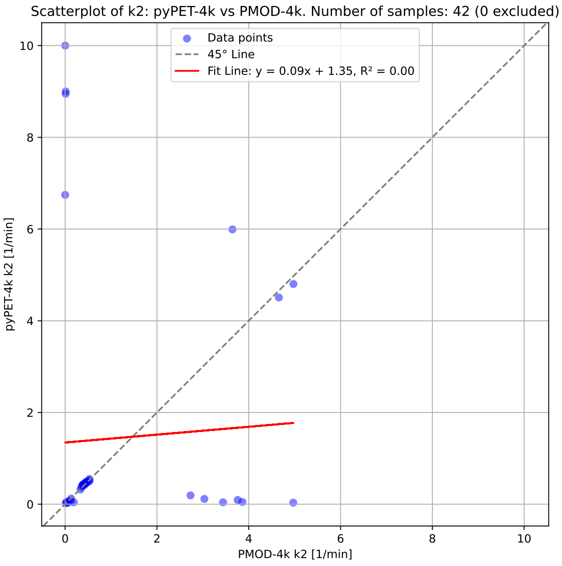

(b)

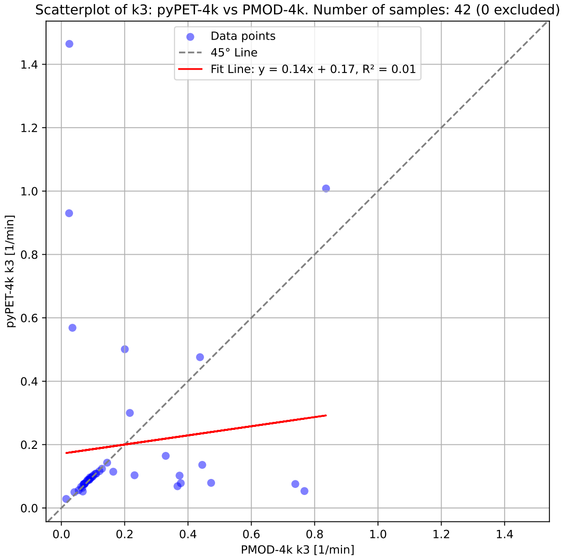

(c)

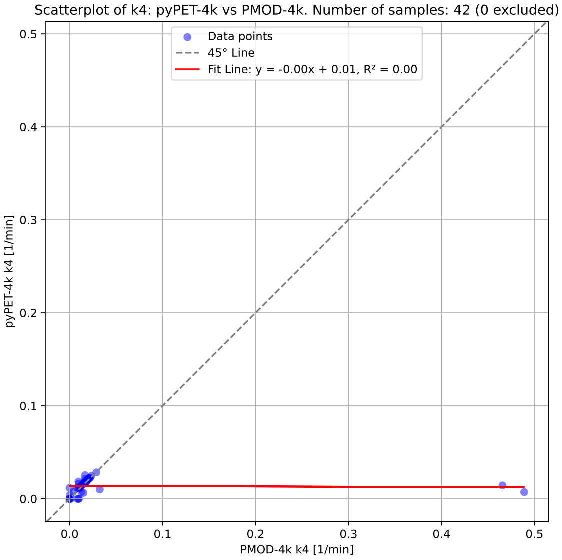

(d)

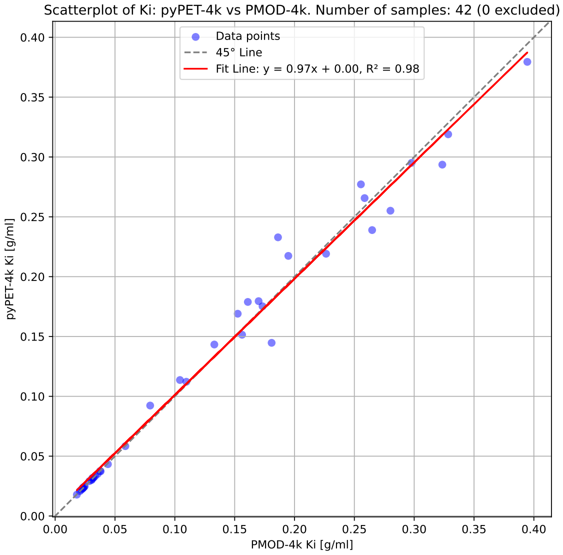

(e)

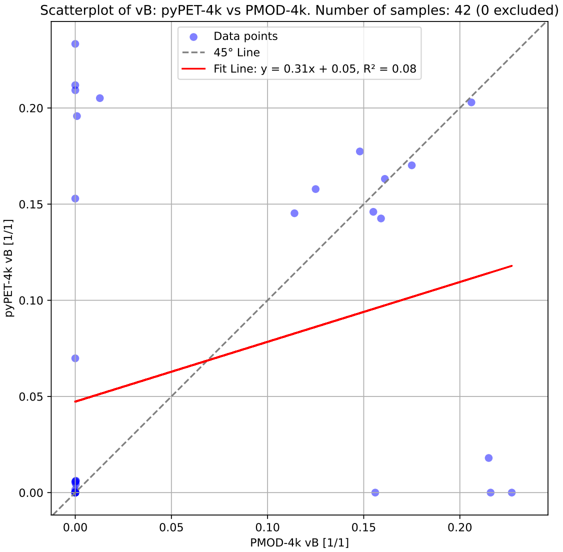

(f)

Figure S9: Comparison of kinetic modeling parameters calculated using from pyPET and PMOD software packages. Subfigures (a) through (f) shows K_1, k_2, k_3, k_4, K_i and vB, respectively. All 42 samples are shown.

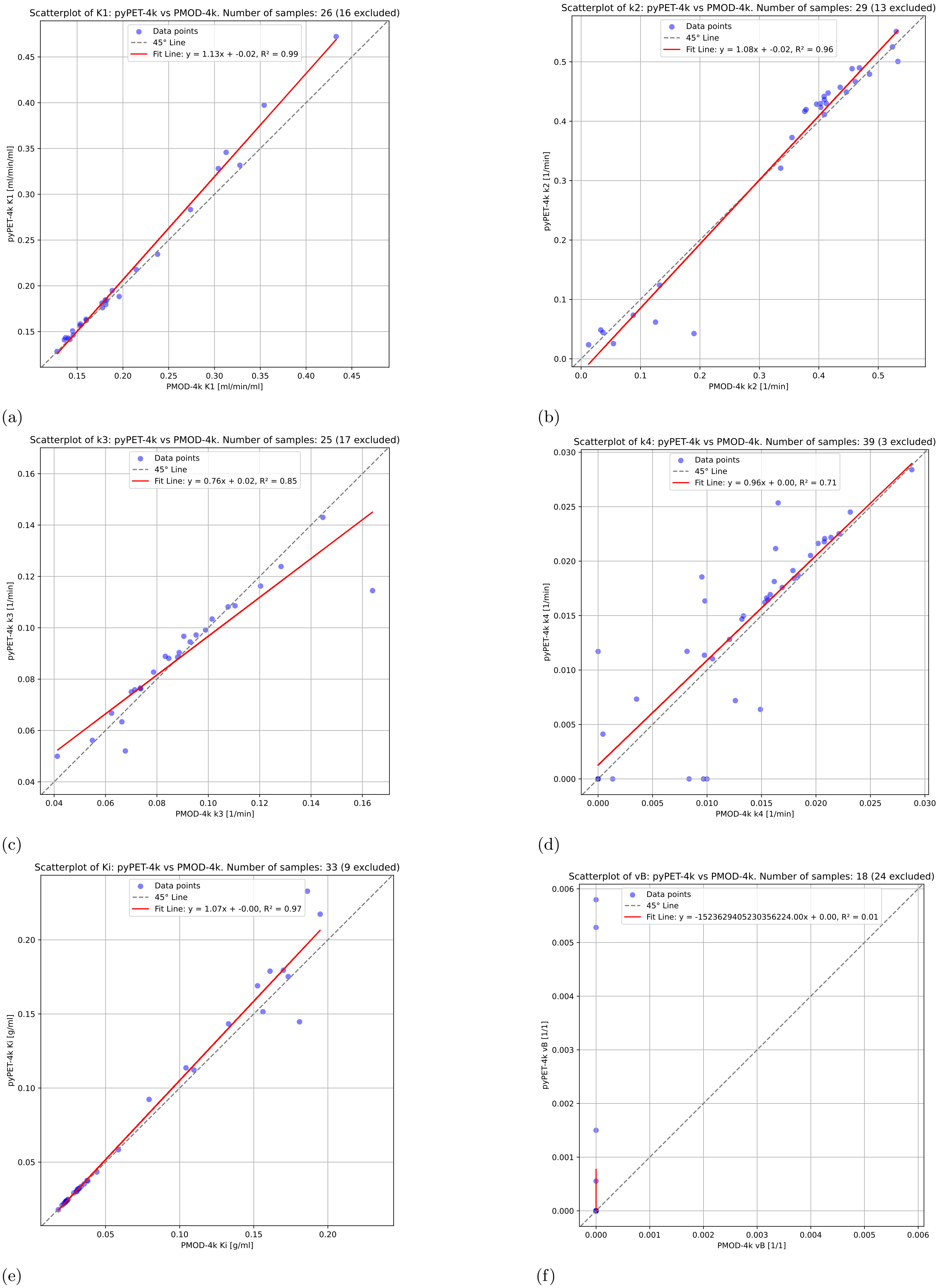

Figure S10: Comparison of kinetic modeling parameters calculated using from pyPET and PMOD software packages. Subfigures (a) through (f) shows K_1, k_2, k_3, k_4, K_i and vB, respectively. Samples with kinetic modeling parameters outside three scaled median absolute deviations from the median of each parameter were considered outliers and removed from the comparison.

References

[1] Alejandro Santillan, David G. Rubin, Conor P. Foley, et al. "Cannulation of the internal carotid artery in mice: A novel technique for intra-arterial delivery of therapeutics". In: *Journal of Neuroscience Methods* 222 (2014), pp. 106–110. ISSN: 01650270. DOI: 10.1016/j.jneumeth.2013.11.008.

[2] Laurence Convert, Otman Sarrhini, Maxime Paillé, et al. "The ultra high sensitivity blood counter: A compact, MRI-compatible, radioactivity counter for pharmacokinetic studies in μl volumes". In: *Biomedical Physics and Engineering Express* 8 (3 May 2022). ISSN: 20571976. DOI: 10.1088/2057-1976/AC4C29.

[3] Geoff Warnock, Mohamed Ali Bahri, David Goblet, et al. "Use of a beta microprobe system to measure arterial input function in PET via an arteriovenous shunt in rats". In: *EJNMMI Research* 1 (1 2011), pp. 1–11. ISSN: 2191219X. DOI: 10.1186/2191-219X-1-13.

[4] Mia Hubert and Stephan Van der Veeken. "Outlier detection for skewed data". In: *J. Chemom.* 22.3-4 (2008), pp. 235–246. ISSN: 08869383. DOI: 10.1002/cem.1123.

[5] Dagan Feng, Sung Cheng Huang, and Xinmin Wang. "Models for computer simulation studies of input functions for tracer kinetic modeling with positron emission tomography". In: *Int. J. Biomed. Comput.* 32.2 (1993), pp. 95–110. ISSN: 00207101. DOI: 10.1016/0020-7101(93)90049-C.

[6] H. Iida, I. Kanno, S. Miura, et al. "Error analysis of a quantitative cerebral blood flow measurement using H215O autoradiography and positron emission tomography, with respect to the dispersion of the input function". In: *J. Cereb. Blood Flow Metab.* 6.5 (1986), pp. 536–545. DOI: 10.1038/jcbfm.1986.99.

[7] Samuel Kuttner, Kristoffer Knutsen Wickstrøm, Gustav Kalda, et al. "Machine learning derived input-function in a dynamic 18 F-FDG PET study of mice". In: *Biomed. Phys. Eng. Express* 6.1 (2020), p. 015020. ISSN: 2057-1976. DOI: 10.1088/2057-1976/ab6496.

[8] Fayçal Ben Bouallègue, Fabien Vauchot, and Denis Mariano-Goulart. "Comparative assessment of linear least-squares, nonlinear least-squares, and Patlak graphical method for regional and local quantitative tracer kinetic modeling in cerebral dynamic 18F-FDG PET". In: *Medical Physics* 46 (3 Mar. 2019), pp. 1260–1271. ISSN: 24734209. DOI: 10.1002/mp.13366.
